# Direct Amidation to Access 3-Amido-1,8-Naphthalimides Including Fluorescent Scriptaid Analogues as HDAC Inhibitors

**DOI:** 10.3390/cells10061505

**Published:** 2021-06-15

**Authors:** Kyle N. Hearn, Trent D. Ashton, Rameshwor Acharya, Zikai Feng, Nuri Gueven, Frederick M. Pfeffer

**Affiliations:** 1 School of Life and Environmental Sciences, Deakin University, Waurn Ponds, VIC 3216, Australia; 2 STEM College, RMIT University, Melbourne, VIC 3000, Australia; kyle.hearn@rmit.edu.au; 3 Walter and Eliza Hall Institute of Medical Research, Parkville, VIC 3052, Australia; ashton.t@wehi.edu.au; 4 Department of Medical Biology, The University of Melbourne, Parkville, VIC 3010, Australia; 5 School of Pharmacy and Pharmacology, College of Health and Medicine, University of Tasmania, Hobart, TAS 7001, Australia; ra26@utas.edu.au (R.A.); zikai.feng@utas.edu.au (Z.F.); nuri.guven@utas.edu.au (N.G.)

**Keywords:** Buchwald–Hartwig, scriptaid, histone deacetylase, HDAC, 1,8-naphthalimide, fluorescence, imaging, tubulin deacetylase

## Abstract

Methodology to access fluorescent 3-amido-1,8-naphthalimides using direct Buchwald–Hartwig amidation is described. The protocol was successfully used to couple a number of substrates (including an alkylamide, an arylamide, a lactam and a carbamate) to 3-bromo-1,8-naphthalimide in good yield. To further exemplify the approach, a set of scriptaid analogues with amide substituents at the 3-position were prepared. The new compounds were more potent than scriptaid at a number of histone deacetylase (HDAC) isoforms including HDAC6. Activity was further confirmed in a whole cell tubulin deacetylation assay where the inhibitors were more active than the established HDAC6 selective inhibitor Tubastatin. The optical properties of these new, highly active, compounds make them amenable to cellular imaging studies and theranostic applications.

## 1. Introduction

Interest in functionalised 1,8-naphthalimides has primarily focussed on substitution at the 4-position to generate fluorophores suitable for sensing and imaging applications [1,2,3,4]. Examples where sensors have been modified in the 3-position are less common, with notable examples including those reported by Zhang et al., Guo et al. and Elmes et al. for the detection of CO_2_, ClO^−^ and reductive stress, respectively [5,6,7]. Examples that incorporate a 3-amido substituent are particularly rare [5,8,9,10], likely due to the multistep nature of the synthetic protocols required to access them. We have recently demonstrated that in the synthesis of 4-amido-1,8-naphthalimides, the usual three-step approach can be avoided using a Buchwald–Hartwig cross-coupling protocol in which a range of amides as well as lactams, carbamates and urea can be introduced in a single step [11]. Nicotinamides were also successfully coupled and the resultant probes shown to act as reversible indicators of the cellular redox state [12]. This direct coupling approach has not yet been evaluated for introducing substituents at the 3-position.

**Figure 1:**
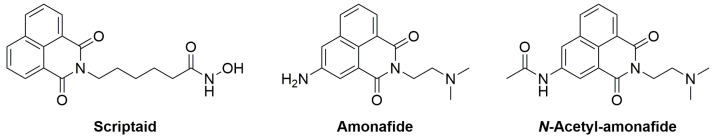
Examples of 1,8-naphthalimides in medicinal chemistry.

The 1,8-naphthalimide core has also been employed in medicinal chemistry [13,14]. Relevant examples (Figure 1) include (i) scriptaid [15] (an inhibitor of histone deacetylases (HDAC)) and amonafide [16,17,18,19] (a DNA intercalator and topoisomerase II inhibitor). Amonafide is converted in vivo into the bioactive *N*-acetyl-amonafide.

The HDAC family has become well-studied due to their roles in a number of disease states [20,21,22]. Indeed, a number of HDAC inhibitors are FDA-approved for clinical use as treatments for T-cell lymphoma or myeloma [23,24,25,26,27]. In an effort to mitigate side effects, the next generation of HDAC inhibitors have been developed to be selective for specific HDAC classes or isoforms [28,29,30]. In this context, HDAC6 (Class IIb) has emerged as a valuable target as it has a clear role in the progression of a number of cancer types, and, unlike other isoforms, mouse models in which this isoform has been deleted are viable [31,32].

The entrance to the HDAC6 active site is slightly larger [33,34] than that of the other isoforms, and therefore a successful strategy for enhancing selectivity for this isoform is the modification of the pharmacophoric capping group. Amides offer not just a means to introduce additional size; they present both an H-bond donor and acceptor to maximise potential interactions with residues at the active site periphery [35,36,37].

**Figure 2:**
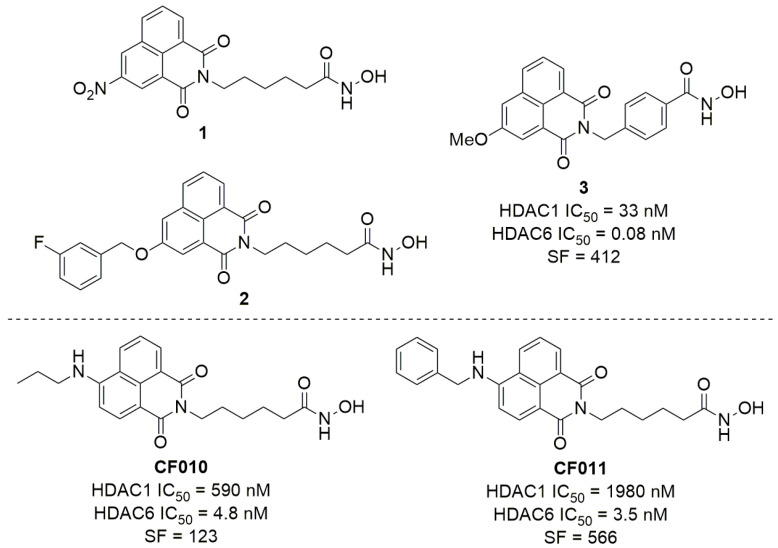
Top: All published examples of 3-substituted scriptaid analogues [38,39,40], and Bottom: selected examples of 4-substituted scriptaid analogues from our recent work [41]. No activity data for **1** and **2** has been published. Selectivity factor (SF) = HDAC1 IC_50_/HDAC6 IC_50_.

There are currently only three published examples of scriptaid analogues with substitution at the 3-position, and only compound **3** has full HDAC IC_50_ activity data recorded (Figure 2) [38,39,40]. There are no scriptaid analogues with 3-amido substituents described in the literature. As such, the generation of a small set of 3-amido-substituted scriptaid analogues presents the opportunity to (i) test the Buchwald–Hartwig amidation methodology beyond the 4-position and to (ii) further explore the structure-activity relationship of functionalised scriptaid analogues. Our own recent work (**CF010** and **CF011**, Figure 2) has identified that, for the 4-position, relatively small substituents can dramatically influence isoform selectivity and fluorescence properties [41]. While our efforts have focussed on modifying scriptaid to develop highly fluorescent anticancer agents, related recent examples exist for identifying biofilms and detecting influenza [42,43].

Herein we outline the methodology for the direct synthesis of 3-amido-1,8-naphthalimides and the use of this approach to construct a set of scriptaid analogues. The results of the photophysical and biological assessment (IC_50_ and in cell tubulin acetylation) are also presented.

## 2. Results and Discussion

### 2.1. Methodology

**Scheme 1:**
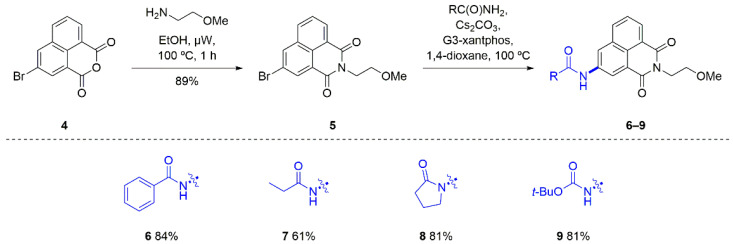
Direct synthesis of 3-amido and 3-carbamato-1,8-naphthalimides.

To trial the Buchwald–Hartwig amidation methodology, 3-bromo-1,8-naphthalimide **4** was first required. The treatment of 1,8-naphthalic anhydride with 1.1 equiv. *N*-bromosuccinimide in H_2_SO_4_ provided the desired brominated compound **4** in >90% purity after trituration with EtOH [44]. The bromoanhydride was then converted to the corresponding methoxyethyl imide **5** using microwave irradiation (Scheme 1, see supplementary material for full details).

Using the reported conditions to introduce amides to the 4-position as a guide [11], a test reaction was performed in which 3-bromo-1,8-naphthalimide **5** was reacted with benzamide, Cs_2_CO_3_ and G3-xantphos at 100 °C (Scheme 1). Monitoring the reaction progress using thin layer chromatography (TLC) indicated the consumption of the starting material and the appearance of a new blue spot (using UV visualisation) within 2.5 h, whereupon the reaction mixture was poured over H_2_O to provide a yellow solid. The analysis of the solid using ^1^H NMR spectroscopy revealed new aryl resonances centred at 8.08 and 7.60 ppm and a new broad singlet at 10.81 ppm that was assigned to the amide N-H proton. Evaluation using HRMS further confirmed that the desired 3-benzamido-1,8-naphthalimide **6** had been synthesised. The product was obtained in good yield (84%) and as such several other substrates—an aliphatic amide (propionamide), a lactam (pyrrolidinone) and a carbamate (*tert*-butyl carbamate)—were also trialled as reaction partners. These additional reactions also provided the desired 3-substituted 1,8-naphthalimide in under 3 h and in good yields (61–81%, Scheme 1). The substrates were well tolerated with yields and reaction times comparable to those observed for the coupling reactions that used 4-bromo-1,8-naphthalimide [11]. In terms of reactivity, 3-bromo-1,8-naphthalimides are an excellent, readily accessible substrate for palladium-mediated amidation reactions.

**Figure 3:**
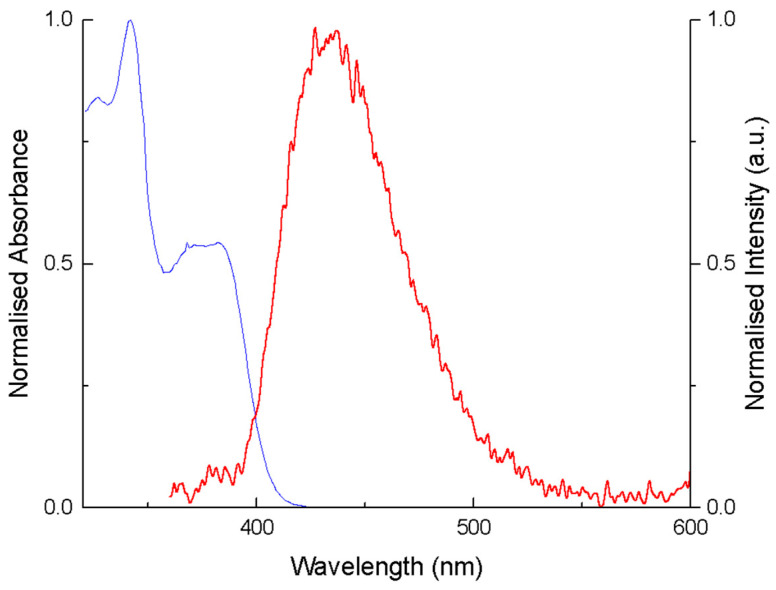
Normalised absorption and emission spectra of **6** in DMSO.

The new compounds showed an absorption maximum and a secondary maximum (~340 and ~380 nm, respectively, Table 1 and Figure 3) and a single emission (~440 nm, Table 1 and Figure 3). Consistent with the decreased extent of intramolecular charge transfer (as compared to the amino substituted examples), the quantum yields for the new compounds ranged from 0.02 to 0.06 (Table 1). For comparison, the 4-benzamido isomer of **6** has Φ_f_ = 0.33 and for the 4-fluorobenzamido Φ_f_ = 0.05 [11]. It is our experience that even with low values the compounds are amenable to imaging applications [45].

### 2.2. Scriptaid Analogues

**Figure 4:**
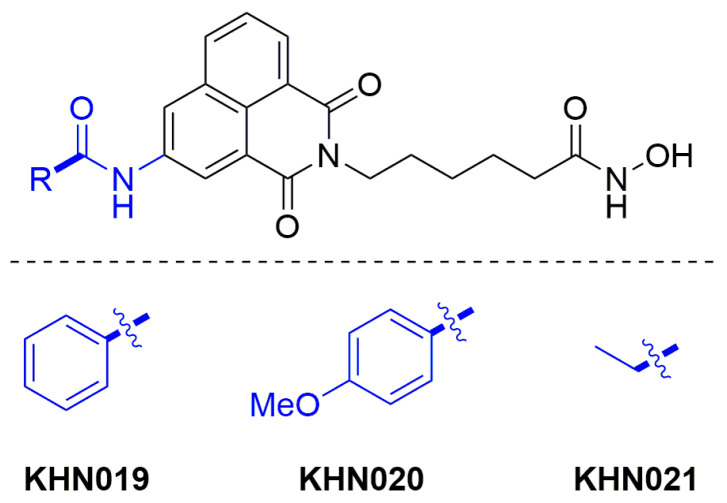
Target 3-amido scriptaid analogues.

The most potent of the previously synthesised 4-amino series were **CF010** and **CF011** (Figure 2) possessing propyl and benzyl substituents, respectively [41]. As such, two aromatic and one aliphatic amide were chosen as suitable substituents for the new 3-amido analogues (**KNH019**, **020** and **021**, respectively, Figure 4).

**Scheme 2:**
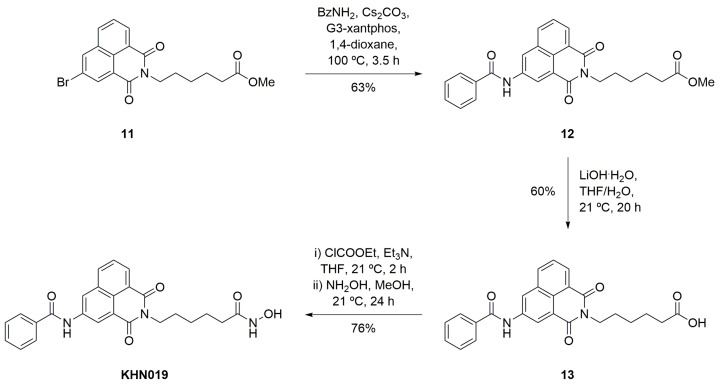
Synthesis of 3-benzamido scriptaid analogue **KNH019**.

The strategy reported by Fleming et al. for the construction of 4-substituted scriptaid analogues was followed here for the synthesis of the 3-substituted relatives [41]. First, 3-bromo-1,8-naphthalic anhydride **4** was transformed into imide **11** in an excellent yield (96% over two steps, see supplementary material for details). Next, the key Buchwald–Hartwig reaction was used to introduce the amido-substituent (Scheme 2). For example, in the synthesis of **KNH019** a solution of 3-bromo-1,8-naphthalimide **11**, benzamide, Cs_2_CO_3_ and G3-xantphos in 1,4-dioxane were heated at 100 °C with the reaction progress being monitored using TLC. Following reaction completion (3.5 h), the desired product **12** was isolated as a light brown solid (63% yield). The methyl ester was carefully removed (1.7 equiv. of LiOH in THF/H_2_O), and the resultant carboxylic acid **13** was converted to the desired hydroxamic acid via the mixed anhydride (formed using ethyl chloroformate and an in-situ treatment with a solution of freshly prepared NH_2_OH in MeOH). After 24 h, the solvent was removed and the residue was triturated with H_2_O to provide the desired hydroxamic acid **KNH019** in a good yield (76% over two steps). In the ^1^H NMR spectrum, two broad singlets at 10.34 and 8.68 ppm (each integrating for one proton) were characteristic of the newly installed hydroxamic acid N-H and O-H protons, respectively. The singlet corresponding to the benzamide N-H proton at 10.85 ppm was also present, indicating the successful formation of the 3-amido scriptaid analogue.

The remaining scriptaid analogues **KNH020** and **KNH021** were accessed in a good yield using the same reaction sequence (see supplementary material for full details). As with the 3-amido-1,8-naphthalimides synthesised at the outset (**6**–**9**), the quantum yield of the new inhibitors ranged from 0.03 to 0.05 (Table 2) with emission at λ_em_ ~437 nm.

### 2.3. HDAC Inhibition

The new scriptaid analogues were initially assessed using a single point assay where enzyme activity is reported as a percentage of full enzyme activity (Table 3). For HDAC isoforms 1, 3, 8 and 11, a concentration of 10 µM of the inhibitor was used. Against HDAC6, inhibition was determined using 0.01 µM of the inhibitor. All 3-amido-1,8-naphthalimide analogues were significantly more effective inhibitors of HDAC6 than the control compound scriptaid. However, no substantial difference in HDAC6 enzyme activity was observed between the benzamide, *p*-methoxybenzamide and propionamide analogues. Furthermore, when the 3-amido scriptaid analogues were evaluated at the “off-target” HDAC isoforms (Table 3), considerable activity was noted; again, they were more effective than scriptaid.

The IC_50_ for the three compounds against the same isoform panel was then measured (Table 4). In agreement with the results of the single point assay, all compounds were potent inhibitors of HDAC6, with similar IC_50_ values (ranging from 0.58 nM to 1.0 nM). Compared to our previously reported 4-amino analogues **CF010** and **CF011 [41]**, the new compounds were more effective inhibitors of HDAC6 (by an order of magnitude) and compared favourably with the 3-methoxy analogue **3** reported by Ho [39]. Based on the inhibition of other isoforms, the selectivity of the new compounds was considered fair to good (ranging from 38 to 150 for HDAC6 over HDAC1); however, when the 3-substituted series were compared to the previous 4-amino analogues **CF010** and **CF011** (up to 566-fold selectivity for HDAC6, Table 4), they were universally less selective. While the set of compounds produced for this study was not extensive, it would appear that amido substitution at the 3-position of the 1,8-naphthalimide core leads to a slightly enhanced activity at all HDAC isoforms to ultimately provide highly potent but only moderately selective HDAC6 inhibitors.

**Figure 5:**
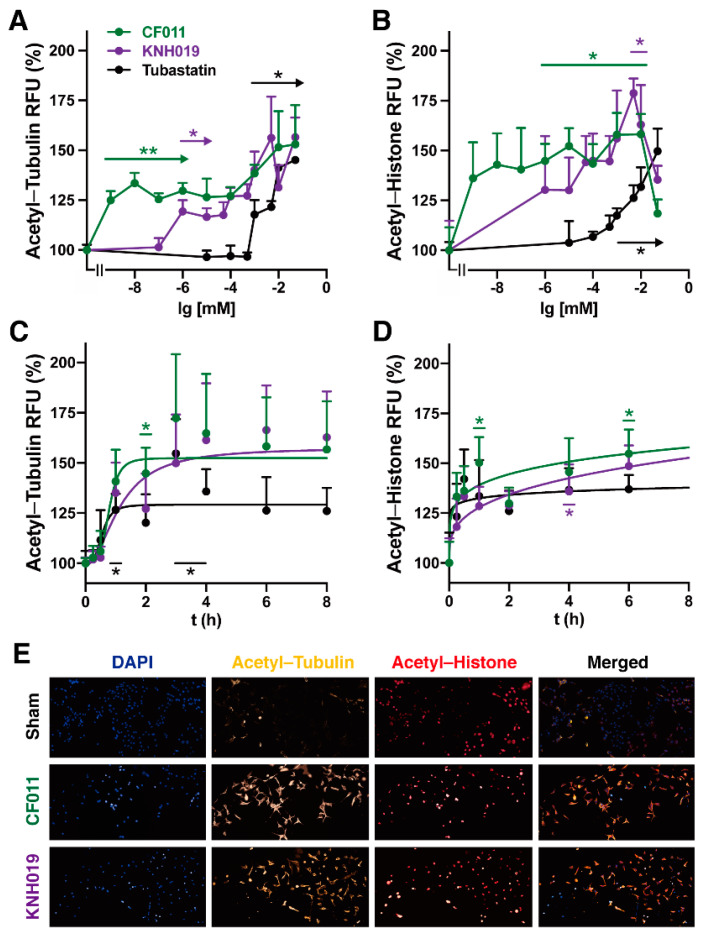
Cell-based assessment of HDAC activity and selectivity. Dose and time-dependency of tubulin and histone acetylation by test compounds. A549 cells were treated with (**A**,**B**) different concentrations (0–50 µM) of **CF011**, **KNH019** and tubastatin for 24 h or with (**C**,**D**) 1 µM over different time intervals before (**A**,**C**) tubulin acetylation or (**B**,**D**) histone acetylation were automatically quantified using high content imaging. Data represent the average of four independent experiments with four replicate wells/experiments. The statistical significances of effects compared to the untreated control were analysed using Brown–Forsythe and Welch one-way ANOVA analyses using Graph Pad Prism. The significance was set as * 0.05 > *p* ≥ 0.01, ** 0.01 > *p* ≥ 0.002. Error bars represent the Standard Error of Mean (SEM). (**E**) Representative fluorescence images show tubulin acetylation (yellow), histone acetylation (red) and nuclear counterstain (DAPI, blue) after 24 h of exposure to test compounds. Secondary antibodies were detected using excitation/emission filters for CY3 (excitation: 542/27 nm, emission: 597/45 nm), CY5 (excitation: 632/22 nm, emission: 684/25 nm) and DAPI (excitation: 390/18 nm, emission: 435/48 nm), respectively.

To confirm the observed activities of the selected test compounds against HDAC6, the acetylation status of tubulin in the human lung cancer cell line A549 was investigated by immunostaining combined with automated high content image analysis (see supplementary material for full details). In addition to the previously published 4-benzylamino scriptaid analogue **CF011** (HDAC6 IC_50_ = 3.5 nM, SF = 566, Table 4), the structurally related 3-benzamido scriptaid **KNH019** (HDAC 6 IC_50_ = 1.0 nM, SF = 91) was tested with regards to dose response and time dependency, where both acetylated tubulin and acetylated histone were detected (Figure 5A–E). Compared to untreated cells, both **CF011** and **KNH019** rapidly increased tubulin acetylation in a dose-dependent manner by up to ∼1.5-fold from 10^−9^ mM and 10^−6^ mM, respectively. In contrast, the reference compound tubastatin only demonstrated a significantly increased tubulin acetylation from 10^−3^ mM in the same test system (Figure 5A). When acetylated histone was detected for both test compounds, **CF011** showed a significant increase from 10^−6^ mM, while **KNH019** only demonstrated a significant activity at 10^−2^ mM. Surprisingly, the reference compound tubastatin increased histone acetylation from 10^−3^ mM, similar to its activity against tubulin acetylation (Figure 5A,B). The apparent drop in histone acetylation by both compounds above 10 μM is indicative of a potential cytotoxicity at higher concentrations (data not shown). Why this effect did not also manifest as reduced tubulin acetylation is unclear at this point.

In A549 cells, all test and reference compounds rapidly increased tubulin and histone acetylation within only a few hours (Figure 5C,D). After 4 h, the increases in tubulin acetylation reached a plateau that was sustained for up to 24 h (data not shown) (Figure 5C,D). Representative immunostaining images also demonstrate that both **CF011** and **KNH019** at 10 µM strongly induce tubulin and histone acetylation. This illustrates that the antibodies used can differentiate between nuclear and cytoplasmic targets and that at this concentration both compounds therefore do not seem to be HDAC6 selective, which predominantly resides in the cytoplasm (Figure 5E). Similar results have been generated in the hepatocarcinoma cell line HepG2 (See supplementary material Figure S24 for details), which highlights that the observed effects are not cell line-specific but are likely relevant for other cells and tissues.

Overall, the data clearly indicate that the new compounds retain HDAC6 activity in cells with a significantly higher potency compared to the reference compound tubastatin. A broader HDAC activity was confirmed for the test and reference compounds when measuring both tubulin and histone acetylation, which reflected the residual activity of isoforms other than HDAC6. The discrepancy between the cell-free and cellular activity observed in this study was reported for tubastatin before and should be considered when using tubastatin as a reference compound [46].

## 3. Conclusions

In summary, the direct amidation of 3-bromo-1,8-naphthalimides was readily achieved using the Buchwald–Hartwig methodology to give amido, lactam and carbamato products. The method was used to produce a set of novel fluorescent scriptaid analogues, and, as identified using direct IC_50_ measurements and whole cell tubulin deacetylation assays, the analogues were potent (but less selective) inhibitors of histone deacetylase enzymes. The fluorescent nature of these compounds makes them well-suited as tools for additional cell-based studies.

## Figures and Tables

**Table 1:** Photophysical properties of 3-amido-1,8-naphthalimides in DMSO.

Compound	λ_abs_ (nm)	λ_em_ (nm)	Stokes Shift (nm)	Φ_f_^†^
**6**	344, 385 ^‡^	435	91, 50	0.02
**7**	341, 383 ^‡^	439	98, 56	0.04
**8**	343, 383 ^‡^	441	98, 58	0.02
**9**	345, 367 ^‡^	442	97, 75	0.06

^†^ Average of two independent samples. ^‡^ Secondary absorption maxima.

**Table 2:** Photophysical properties of 3-amido scriptaid analogues in DMSO.

Compound	λ_abs_ (nm)	λ_em_ (nm)	Stokes Shift (nm)	Φ_f_^†^
**KNH019**	343, 386 ^‡^	437	94, 51	0.03
**KNH020**	343, 374 ^‡^	437	94, 63	0.05
**KNH021**	341, 383 ^‡^	435	94, 54	0.04

^†^ Average of two independent samples. ^‡^ Secondary absorption maxima.

**Table 3:** HDAC activity (percentage of full enzyme activity) for 3-amido scriptaid analogues.

	Average Enzyme Isoform Activity (%) ^†^ HDAC6: 0.01 μM; HDAC1 3, 8, 11: 1.0 μM				
Compound	HDAC1	HDAC3	HDAC6	HDAC8	HDAC11
**scriptaid**	43.2%	43.8%	39.8%	60.3%	78.6%
**KNH019**	36.3%	24.1%	20.4%	69.3%	67.5%
**KNH020**	38.7%	17.2%	19.7%	93.4%	58.7%
**KNH021**	29.8%	20.1%	20.3%	50.4%	90.7%

^†^ Average of two independent measurements, margin of error ±2% (see supplementary material for details).

**Table 4:** HDAC activity (IC_50_) and isoform selectivity factor for 3-amido scriptaid analogues.

	Isoform IC_50_ (μM) ^†^ and Selectivity Factor (SF) against HDAC6				
Compound	HDAC1	HDAC3	HDAC6	HDAC8	HDAC11
**scriptaid**	1.74 ± 0.04 (145)	0.37 ± 0.04 (31)	0.012 ± 0.002	1.52 ± 0.007 (127)	0.36 ± 0.02 (30)
**CF010**	0.59 ± 0.02 (123)	0.11 ± 0.004 (23)	0.0048 ± 0.0002	1.52 ± 0.08 (317)	0.08 ± 0.03 (16)
**CF011**	1.98 ± 0.04 (566)	0.36 ± 0.0007 (103)	0.0035 ± 0.0002	2.46 ± 0.11 (703)	0.15 ± 0.02 (43)
**KNH019**	0.091 ± 0.006 (91)	0.064 ± 0.0002 (64)	0.0010 ± 0.000005	2.95 ± 0.46 (2950)	0.29 ± 0.035 (290)
**KNH020**	0.087 ± 0.007 (150)	0.027 ± 0.003 (47)	0.00058 ± 0.000002	4.33 ± 0.39 (7466)	0.24 ± 0.011 (413)
**KNH021**	0.037 ± 0.002 (38)	0.032 ± 0.001 (332)	0.00097 ± 0.00005	0.95 ± 0.05 (981)	2.16 ± 0.14 (2234)

^†^ Average of two independent measurements (see supplementary material for details).

## Data Availability

All data has been presented in this article or associated supplementary material.

## Supplementary Materials

The following are available online at https://www.mdpi.com/article/10.3390/cells10061505/s1, Pages S2–S11: Experimental procedures, Figures S1–S16: ^1^H and ^13^C NMR of synthesised compounds, Table S1: Photophysical properties, Figures S17–S24: UV/Vis and fluorescence data, Figure S24: Cell-based assessment of HDAC activity and selectivity in HepG2 cells.
