# Supplementary material for: Direct Amidation to Access 3-Amido-1,8-Naphthalimides Including Fluorescent Scriptaid Analogues as HDAC Inhibitors
Source: Cells. 2021 Jun 15;10(6):1505. doi: 10.3390/cells10061505 (PMC8232238; doi:10.3390/cells10061505)
Supplement: Supplementary file 1 [file cells-10-01505-s001.zip › cells-1237189-supplementary.pdf]

Electronic Supplementary Information

Direct amidation to access 3-amido-1,8-naphthalimides including fluorescent scriptaid analogues as HDAC inhibitors

Kyle N. Hearn,[a,b] Trent D. Ashton,[a,c,d] Rameshwor Acharya,[e] Zikai Feng,[e] Nuri Guven[e] and Fred M. Pfeffer[a*]

a School of Life and Environmental Sciences, Deakin University, Waurn Ponds, VIC. 3216, Australia.

b STEM College, RMIT University, Melbourne, VIC. 3000, Australia.

c Walter and Eliza Hall Institute of Medical Research, Parkville, VIC. 3052, Australia.

d Department of Medical Biology, The University of Melbourne, Parkville, VIC. 3010, Australia.

e School of Pharmacy and Pharmacology, College of Health and Medicine, University of Tasmania, Hobart, 7001, Australia

E-mail: fred.pfeffer@deakin.edu.au

Contents:

S1 Chemistry P2

S1.1 General Information P2

S1.2 General Procedure for Palladium-mediated Amidation P2

S1.3 Compound Synthesis P3

S1.4 References P11

S1.5 NMR Spectra P12

S1.6 UV/Vis and Fluorescence Data P28

S2 Biology P33

S2.1 HDAC Assay P33

S2.2 Cell Culture, Treatment and Immunostaining P33

S2.3 Image Analysis P33

S2.4 Cell-based Assessment of HDAC Activity and Selectivity in HepG2 Cells P34

S1 Chemistry

S1.1 General Information

Reactions were monitored using thin-layer chromatography (TLC) with Merck 60 F254 silica gel plates and visualised under UV light at 254 nm. Column chromatography was performed on silica gel 60 (70–230 mesh). Reactions employing microwave irradiation were performed using a CEM Discover S-Class Automated Microwave Sample Preparation System. These reactions were conducted in either 10 mL or 35 mL microwave vials sealed with a snap cap. NH_2OH was freshly prepared in MeOH (0.5 M) using 1:1 ratio of $NH_2OH{\cdot}HCl$ and KOH. G3-Xantphos was prepared as described previous[1].

High resolution mass spectrometry (ESI) was performed using either (i) Applied Biosystems QTOF-MS with a mobile phase of HPLC methanol and 0.1% formic acid or (ii) AB SCIEX TripleTOF 5600 mass spectrometer in a 95% MeOH in H_2O solvent system containing 0.1% formic acid. Analyte solutions were prepared in HPLC grade methanol (conc. ~ 1 mg mL^{-1}).

^{1}H and ^{13}C NMR were performed on either a Bruker Ascend III 300 MHz, Bruker Ascend III 400 MHz or Bruker Ascend III 500 MHz spectrometer. Samples were dissolved in CDCl3 (^{1}H δ 7.26, ^{13}C δ 77.16) or DMSO-*d*6 (^{1}H δ 2.50, ^{13}C δ 39.52) and are reported in reference to the residual solvent signal. ^{1}H NMR spectra are reported as: chemical shift δ (ppm), multiplicity (s singlet, d doublet, t triplet, dd doublet of doublets, m multiplet, dt doublet of triplets, td triplet of doublets), *J* coupling (reported in Hz), equivalent nuclei.

S1.2 General Procedure for Palladium-mediated Amidation

An oven dried flask, equipped with a stirrer bar was cooled under N_2 and charged with 3-bromo-1,8-naphthalimide, the requisite amide, carbamate or urea (1.2–2.0 equiv), Cs_2CO_3 (1.4 equiv), G3-Xantphos (0.01 equiv) and 1,4-dioxane (5 mL per 0.5 mmol of aryl halide). The resultant mixture was heated at 100 °C for the indicated time. At the completion of the reaction H_2O was added to provide a precipitate that was collected using vacuum filtration and washed using H_2O. Further purification (if required) was performed as specified.

S1.3 Compound Synthesis

5-Bromo-1*H*,3*H*-benzo[*de*]isochromene-1,3-dione (4)

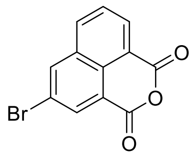

A stirring solution of 1,8-naphthalic anhydride (833 mg, 4.20 mmol) and H_2SO_4 (17 mL) was charged with *N*-bromosuccinimide (774 mg, 4.35 mmol, 1.1 equiv) and allowed to stir at ambient temperature for 18 h. This solution was then added dropwise into H_2O (105 mL) while stirring at 0 °C. The precipitate was collected using vacuum filtration and the crude residue was crystallised from DMSO to provide the desired compound in >90% purity as a white solid (404 mg, 40%).

^{1}H NMR (DMSO-d_6, 500 MHz): δ 8.86 (d, *J* = 1.9 Hz, 1H), 8.54 (dd, *J* = 7.3, 1.0), 8.51–8.48 (m, 2H), 7.96 (dd, *J* = 8.1, 7.3, 1H). Data consistent with the literature[2].

^{13}C NMR (DMSO-d_6, 125 MHz): δ 160.2, 159.7, 136.8, 134.4, 134.0, 132.7, 132.6, 128.8, 128.4, 121.5, 120.1, 119.5. Data consistent with the literature[2].

5-Bromo-2-(2-methoxyethyl)-1*H*-benzo[*de*]isoquinoline-1,3(2*H*)-dione (5)

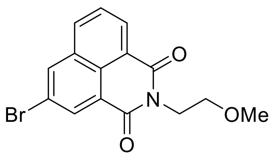

A 10 mL microwave vial charged with 3-bromo-1,8-naphthalic anhydride **4** (408 mg, 1.47 mmol), 2-methoxyethylamine (140 µL, 1.62 mmol, 1.1 equiv) and EtOH (4.5 mL) was heated using microwave irradiation for 1 h at 100 °C. The reaction mixture was diluted with H_2O (20 mL) and the resultant solid was collected using vacuum filtration. The crude solid was purified using column chromatography (15% EtOAc/pet. spirits) to afford the title compound in >95% purity as a yellow solid (438 mg, 89%).

^{1}H NMR (300 MHz, $CDCl_3$): δ 8.66 (d, *J* = 1.9, 1H), 8.60 (dd, *J* = 1.0, 7.4, 1H), 8.36 (d, *J* = 1.9, 1H), 8.12 (d, *J* = 1.0, 8.2, 1H), 7.77 (dd, *J* = 7.4, 8.2, 1H), 4.44 (t, *J* = 5.8, 2H), 3.73 (t, *J* = 5.8, 2H), 3.37 (s, 3H).

^{13}C NMR (75 MHz, DMSO-d_6): δ 163.9, 163.3, 135.7, 134.3, 133.0 (2C), 131.7, 128.2, 126.9, 124.3, 123.0, 121.3, 69.7, 59.0, 39.6*.

HRMS (ESI) for $C_{15}H_{12}BrNO_3$ [M + H$^+$] calcd, 334.0074; found, 334.0068.

*Determined using HSQC

***N*-(2-(2-Methoxyethyl)-1,3-dioxo-2,3-dihydro-1*H*-benzo[*de*]isoquinolin-5-yl)benzamide (6)**

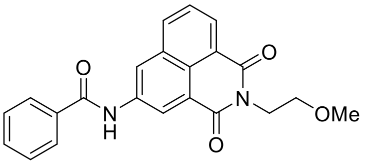

Following the general procedure, 3-bromo-1,8-naphthalimide **5** (86 mg, 0.248 mmol), benzamide (36 mg, 0.298 mmol, 1.2 equiv), G3-Xantphos (2.4 mg, 0.0025 mmol, 0.01 equiv), Cs_2CO_3 (114 mg, 0.349 mmol, 1.4 equiv) and 1,4-dioxane (2.5 mL) were reacted at 100 °C for 135 min. Provide the title compound in >95% purity as an orange solid (78 mg, 84%).

^{1}H NMR ($CDCl_3$, 300 MHz): δ 9.07 (d, *J* = 1.2, 1H), 8.49 (d, *J* = 7.2, 1H), 8.37 (d, *J* = 2.2, 1H), 8.33 (br. s, 1H), 8.18 (d, *J* = 8.3, 1H), 7.98–7.95 (m, 2H), 7.75–7.70 (m, 1H), 7.65–7.53 (m, 3H), 4.43 (t, *J* = 5.7, 2H), 3.74 (t, *J* = 5.7, 2H), 3.38 (s, 3H).

^{13}C NMR ($CDCl_3$, 75 MHz): δ 166.2, 164.3, 164.1, 136.8, 134.4, 134.1, 132.7, 132.6, 130.4, 129.2, 127.8, 127.3, 125.4, 124.5, 123.5, 122.8, 122.5, 69.8, 58.9 39.5.

HRMS (ESI) for $C_{22}H_{18}N_2O_4$ [M + H$^+$] calcd, 375.1340; found, 375.1329.

***N*-(2-(2-Methoxyethyl)-1,3-dioxo-2,3-dihydro-1*H*-benzo[*de*]isoquinolin-5-yl)propionamide (7)**

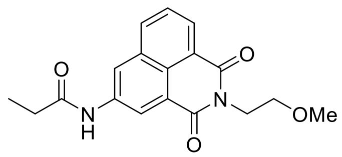

Following the general procedure, 3-bromo-1,8-naphthalimide **5** (86 mg, 0.246 mmol), propionamide (22 mg, 0.299 mmol, 1.2 equiv), G3-Xantphos (4.1 mg, 0.004 mmol, 0.01 equiv), Cs_2CO_3 (131 mg, 0.401 mmol, 1.6 equiv) and 1,4-dioxane (2.5 mL) were reacted at 100 °C for 75 min. The crude material was recrystallised from EtOH to provide the desire product in >95% purity as a yellow solid (49 mg, 61%).

^{1}H NMR ($CDCl_3$, 500 MHz): δ 8.80 (s, 1H), 8.42 (d, *J* = 7.2, 1H), 8.12 (d, *J* = 1.5, 1H), 8.03 (d, *J* = 8.2, 1H), 7.87 (s, 1H), 7.67 (t, 7.7, 1H), 4.44 (t, *J* = 5.6, 2H), 3.80 (t, *J* = 5.6, 2H), 3.43 (s, 3H), 2.52 (q, *J* = 7.5, 2H), 1.32 (t, *J* = 7.5, 3H).

^{13}C NMR ($CDCl_3$, 125 MHz): δ 172.9, 164.4, 164.0, 136.7, 134.0, 132.6, 130.1, 127.6, 124.9, 123.8, 123.1, 122.2, 122.0, 70.0, 58.9, 39.5, 30.9, 9.6.

HRMS (ESI) for $C_{18}H_{18}N_2O_4$ [M + H$^+$] calcd, 327.1339; found, 327.1347.

2-(2-Methoxyethyl)-5-(2-oxopyrrolidin-1-yl)-1*H*-benzo[*de*]isoquinoline-1,3(2*H*)-dione (8)

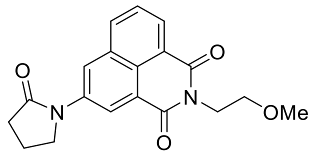

Following the general procedure, 3-bromo-1,8-naphthalimide **5** (87 mg, 0.250 mmol), pyrrolidinone (20 µL, 0.263 mmol, 1.1 equiv), G3-Xantphos (2.4 mg, 0.0025 mmol, 0.01 equiv), Cs_2CO_3 (117 mg, 0.359 mmol, 1.4 equiv) and 1,4-dioxane (2.5 mL) were reacted at 100 °C for 100 min. The reaction mixture was diluted with H_2O (20 mL), transferred to a separatory funnel and extracted into CH_2Cl_2 (2 × 25 mL). Organic extracts were combined, washed with brine (20 mL), dried, filtered and solvent removed under reduced pressure to provide the title compound in >95% purity as a yellow oil (68 mg, 81%).

^{1}H NMR ($CDCl_3$, 300 MHz): δ 8.86 (d, *J* = 2.2, 1H), 8.59 (d, *J* = 2.2, 1H), 8.52 (dd, *J* = 7.3, 1.0, 1H), 8.20–8.17 (dd, *J* = 8.2, 1.0, 1H), 7.73 (dd, *J* = 8.2, 7.3, 1H), 4.45 (t, *J* = 5.8, 2H), 4.08 (t, *J* = 7.0, 2H), 3.73 (t, *J* = 5.8, 2H), 3.38 (s, 3H), 2.72 (t, *J* = 8.1, 2H), 2.33–2.22 (m, 2H).

^{13}C NMR ($CDCl_3$. 75 MHz): δ 174.9, 164.4, 164.2, 138.2, 134.1, 132.4, 130.6, 127.6, 125.3, 123.5, 123.4, 123.3, 122.5, 69.8, 59.0, 48.9, 39.5, 32.9, 18.1.

HRMS (ESI) for $C_{19}H_{18}N_2O_4$ [M + H$^+$] calcd, 339.1340; found, 339.1340.

***tert*-Butyl (2-(2-methoxyethyl)-1,3-dioxo-2,3-dihydro-1*H*-benzo[*de*]isoquinolin-5-yl)carbamate (9)**

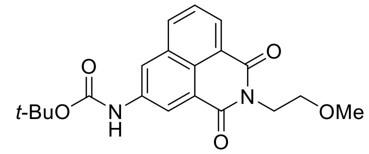

Following the general procedure, 3-bromo-1,8-naphthalimide **5** (87 mg, 0.249 mmol), *tert*-butyl carbamate (35 mg, 0.300 mmol, 1.2 equiv), G3-Xantphos (2.5 mg, 0.0026 mmol, 0.01 equiv), Cs_2CO_3 (114 mg, 0.350 mmol, 1.4 equiv) and 1,4-dioxane (2.5 mL) were reacted at 100 °C for 2.5 h. Provides the title compound in >95% purity as a yellow solid (75 mg, 81%).

^{1}H NMR ($CDCl_3$, 300 MHz): δ 8.55 (br. s, 1H), 8.42 (dd, *J* = 7.3, 1.0, 1H), 8.22 (d, *J* = 2.2, 1H), 8.05 (d, *J* = 7.7, 1H), 7.69–7.64 (m, 1H), 7.21 (s, 1H), 4.45 (t, *J* = 5.7, 2H), 3.77 (t, *J* = 5.7, 2H), 3.41 (s, 3H), 1.58 (s, 9H).

^{13}C NMR ($CDCl_3$, 75 MHz): δ 164.4, 164.1, 152.8, 137.5, 133.6, 132.8, 129.6, 127.5, 124.6, 123.7, 123.2, 122.3, 120.0, 81.6, 69.8, 58.9, 39.3, 28.5.

HRMS (ESI) for $C_{20}H_{22}N_2O_5$ [M + H$^+$] calcd, 371.1602; found, 371.1602.

6-(5-Bromo-1,3-dioxo-1*H*-benzo[*de*]isoquinolin-2(3*H*)-yl)hexanoic acid (10)

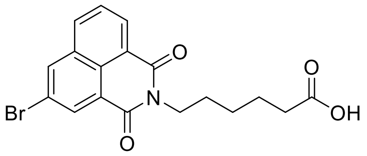

A 30 mL microwave vial charged with 3-bromo-1,8-naphthalic anhydride (1.115 g, 4.026 mmol), 6-aminohexanoic acid (606 mg, 4.622 mmol, 1.1 equiv) and EtOH (10 mL) was heated using microwave irradiation at 100 °C for 1 h. The reaction mixture was diluted with H_2O (50 mL) and the resultant solid was collected using vacuum filtration. Provides the title compound (1.522 g, 97%) as a white solid that was used without further purification.

^{1}H NMR (DMSO-d_6, 400 MHz): δ 8.74 (d, *J* = 1.9, 1H), 8.48 (dd, *J* = 7.3, 1.0, 1H), 8.42 (d, *J* = 1.9, 1H), 8.39 (dd, *J* = 8.3, 1.0, 1H), 7.90 (dd, *J* = 8.3, 7.4, 1H), 4.00 (t, *J* = 7.3. 2H). 2.20 (t, *J* = 7.3, 2H), 1.66–1.50 (m, 4H), 1.38–1.30 (m, 2H).

^{13}C NMR (DMSO-d_6, 75 MHz): δ 174.5, 163.0, 162.4, 135.7, 133.4, 132.7, 132.5, 131.1, 128.4, 126.0, 124.1, 122.3, 120.0, 39.3*, 33.5, 27.2, 26.0, 24.2.

HRMS (ESI) for $C_{18}H_{16}BrNO_4$ [M + H$^+$] calcd, 404.0492; found, 404.0494.

*Determined using HSQC

Methyl 6-(5-bromo-1,3-dioxo-1*H*-benzo[*de*]isoquinolin-2(3*H*)-yl)hexanoate (11)

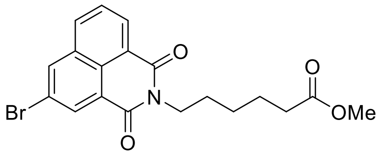

A stirring solution of 3-bromo-1,8-naphthalimide **10** (912 mg, 2.34 mmol), MeOH (20 mL) and H_2SO_4 (10 drops) was heated at 65 °C for 48 h. After cooling to ambient temperature, the reaction mixture was diluted with H_2O (40 mL) and the resulting white precipitate was collected using vacuum filtration to provide the desired product (899 mg, 95%) in >90% purity as a white solid which was used without further purification.

^{1}H NMR ($CDCl_3$, 500 MHz): δ 8.64 (d, *J* = 6.0, 1H), 8.59 (d, *J* = 7.3, 1H), 8.36 (d, *J* = 6.0, 1H), 8.12 (d, *J* = 8.3, 1H), 7.81 (t, *J* = 7.8, 1H), 4.18–4.15 (m, 2H), 3.66 (s, 3H), 2.33 (t, *J* = 7.5, 2H), 1.77–1.68 (m, 4H), 1.48–1.42 (m, 2H).

^{13}C NMR (DMSO-d_6, 100 MHz): δ 173.4, 163.0, 162.4, 135.7, 133.4, 132.7, 132.5, 131.1, 128.5, 126.0, 124.1, 122.3, 120.1, 51.2, 39.8* 33.1, 27.1, 25.9, 24.2.

HRMS (ESI) for $C_{19}H_{18}BrNO_4$ [M + H$^+$] calcd, 404.0492; found, 404.0494.

*Determined using HSQC

Methyl 6-(5-benzamido-1,3-dioxo-1*H*-benzo[*de*]isoquinolin-2(3*H*)-yl)hexanoate (12)

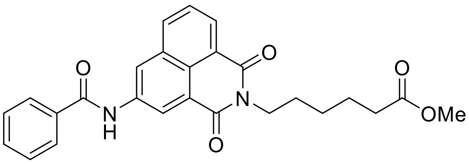

Using the general procedure, 3-bromo-1,8-naphthalimide **11** (208 mg, 0.515 mmol), benzamide (120 mg, 0.987 mmol, 1.9 equiv), G3-Xantphos (4.9 mg, 0.005 mmol, 0.01 equiv), Cs_2CO_3 (272 mg, 0.835 mmol, 1.6 equiv) in 1,4-dioxane (5.0 mL) were allowed to react at 100 °C for 3.5 h. The crude solid was recrystallised using EtOH to provide the title compound as a fluffy brown solid (143.9 mg, 63%).

^{1}H NMR (DMSO-d_6, 400 MHz): δ 10.84 (s, 1H), 9.01 (d, *J* = 1.9, 1H), 8.87 (d, *J* = 1.9, 1H), 8.42 (d, *J* = 8.4, 1H), 8.39 (d, *J* = 7.4, 1H), 8.07 (d, *J* = 7.1, 2H), 7.84 (t, *J* = 7.8, 1H), 7.67–7.63 (m, 1H), 7.61–7.57 (m, 2H), 4.04 (t, *J* = 7.3, 2H), 3.57 (s, 3H), 2.32 (t, *J* = 7.3, 2H), 1.68–1.55 (m, 4H), 1.39–1.32 (m, 2H).

^{13}C NMR ($CDCl_3$, 125 MHz): δ 174.3, 166.4, 164.2, 164.0, 136.9, 134.3, 134.0, 132.7, 132.5, 130.2, 129.1, 127.7, 127.4, 125.3, 124.6, 123.4, 122.9, 122.4, 51.7, 40.4, 34.1, 27.8, 26.7, 24.8.

HRMS (ESI) for $C_{26}H_{24}N_2O_5$ [M + H$^+$] calcd, 445.1758; found, 445.1755.

6-(5-Benzamido-1,3-dioxo-1*H*-benzo[*de*]isoquinolin-2(3*H*)-yl)hexanoic acid (13)

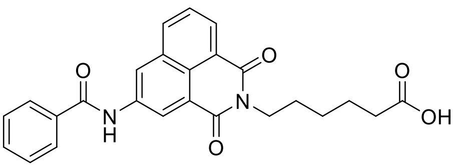

A stirring solution of methyl ester **12** (118 mg, 0.266 mmol) and THF/H_2O (1:1, 10 mL) was treated with LiOH·H_2O (20 mg, 0.465 mmol, 1.7 equiv) at ambient temperature for 20 h. After this time, the solution volume was reduced to half using a rotary evaporator and diluted to 15 mL with H_2O. The solution was acidified to pH 1 with 1 M HCl, transferred to a separatory funnel and the aqueous phase was extracted with 10% MeOH in CH_2Cl_2 (3 × 40 mL). Organic extracts were combined, dried, filtered and solvent removed under reduced pressure to provide the title compound as a yellow solid that was used without further purification (68.6 mg, 60%).

^{1}H NMR (DMSO-d_6, 400MHz): δ 10.85 (s, 1H), 9.02 (d, *J* = 7.9, 1H), 8.88 (d, *J* = 1.9, 1H), 8.43 (d, *J* = 8.3, 1H), 8.40 (d, *J* = 7.6, 1H), 8.08 (d, *J* = 7.2, 2H), 7.85 (t, *J* = 7.8, 1H), 7.66–7.58 (m, 3H), 4.05 (t, *J* = 7.3, 2H), 2.22 (t, *J* = 7.3, 2H), 1.67–1.52 (m, 4H), 1.40–1.32 (m, 2H).

^{13}C NMR (DMSO-d_6, 75 MHz): δ 174.5, 166.1, 163.5, 163.3, 138.1, 134.3, 133.9, 132.1, 132.0, 129.2, 128.6, 127.9, 127.7, 125.0, 124.3, 122.6, 122.1, 121.9, 39.1, 33.5, 27.3, 26.1, 24.3.

HRMS (ESI) for $C_{25}H_{22}N_2O_5$ [M + H$^+$] calcd, 431.1601; found, 431.1598.

N-(2-(6-(Hydroxyamino)-6-oxohexyl)-1,3-dioxo-2,3-dihydro-1*H*-benzo[*de*]isoquinolin-5-yl)benzamide (KNH019)

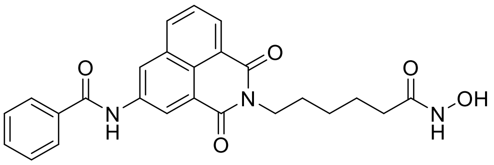

A stirring solution of carboxylic acid **13** (64 mg, 0.148 mmol) and THF (3 mL) was charged with Et_3N (40 µL, 0.287 mmol, 1.9 equiv) and $ClCO_2Et$ (15 µL, 0.158 mmol, 1.1 equiv) and stirred for 4 h at ambient temperature. The solution was filtered to remove $Et_3N{\cdot}HCl$ and the filtrate was directly added to a freshly prepared solution of NH_2OH in MeOH (0.5 M, 3.0 mL) before stirring for 24 h at room temperature. Excess solvent was removed *in vacuo* and the crude residue was triturated using H_2O to provide the title compound as a pale yellow solid (50 mg, 76%).

^{1}H NMR (DMSO-d_6, 400 MHz): δ 10.85 (s, 1H), 10.33 (s, 1H), 9.01 (d, *J* = 1.8, 1H), 8.88 (d, *J* = 1.8, 1H), 8.67 (s, 1H), 8.42 (d, *J* = 8.2, 1H), 8.40 (d, *J* = 7.3, 1H), 8.07 (d, *J* = 7.3, 2H), 7.84 (t, *J* = 7.8, 1H), 7.65–7.57 (m, 3H), 4.04 (t, *J* = 7.3, 2H), 1.96 (t, *J* = 7.3, 2H), 1.67–1.51 (m, 4H), 1.36–1.28 (m, 2H).

^{13}NMR (DMSO-d_6, 100 MHz): δ 169.1, 166.1, 163.5, 163.3, 138.1, 134.3, 133.9, 132.1, 129.2, 128.6, 127.9, 127.7, 125.1, 125.0, 124.3, 122.7, 122.2, 121.9, 39.9*, 32.2, 27.0, 26.2, 24.9.

HRMS (ESI) for $C_{25}H_{23}N_3O_5$ [M + H$^+$] calcd, 446.1710; found, 446.1716.

*Determined using HSQC

Methyl 6-(5-(4-methoxybenzamido)-1,3-dioxo-1*H*-benzo[*de*]isoquinolin-2(3*H*)-yl)hexanoate (14)

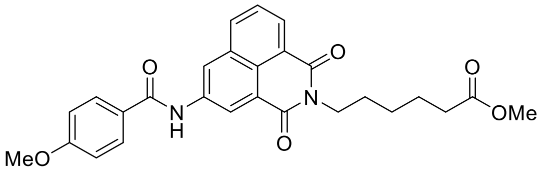

Using the general procedure, 3-bromo-1,8-naphthalimide **11** (199 mg, 0.492 mmol), benzamide (111 mg, 0.734 mmol, 1.5 equiv), G3-Xantphos (4.6 mg, 0.005 mmol, 0.01 equiv), Cs_2CO_3 (331 mg, 1.015 mmol, 2.1 equiv) in 1,4-dioxane (5.0 mL) were allowed to react at 100 °C for 105 min. Provides the title compound as a yellow solid that was used without further purification (205.2 mg, 88%).

^{1}H NMR (DMSO-d_6, 400 MHz): δ 10.64 (s, 1H), 8.96 (d, *J* = 1.8, 1H), 8.84 (d, *J* = 1.8, 1H), 8.37–8.34 (m, 2H), 8.06 (d, *J* = 8.8, 2H), 7.80 (t, *J* = 7.8, 1H), 7.10 (d, *J* = 8.8, 2H), 4.02 (t, *J* = 7.3, 2H), 3.86 (s, 3H), 3.56 (s, 3H), 2.31 (t, *J* = 7.3, 2H), 1.63–1.54 (m, 4H), 1.38–1.32 (m, 2H).

^{13}C NMR (DMSO-d_6, 100 MHz): δ 173.4, 165.4, 163.5, 163.3, 162.3, 138.3, 133.9, 132.1, 129.9, 129.1, 127.6, 126.3, 125.1, 124.2, 122.6, 121.9, 121.9, 113.8, 55.5, 51.2, 39.5*, 33.1, 27.2, 26.0, 24.2.

HRMS (ESI) for $C_{27}H_{26}N_2O_6$ [M + H$^+$] calcd, 475.1864; found 475.1868

*Determined using HSQC

6-(5-(4-Methoxybenzamido)-1,3-dioxo-1*H*-benzo[*de*]isoquinolin-2(3*H*)-yl)hexanoic acid (15)

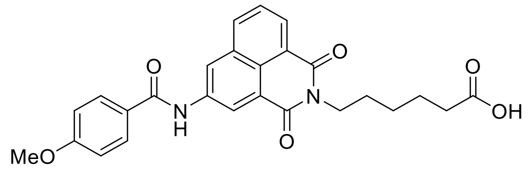

A stirring solution of methyl ester **14** (174 mg, 0.366 mmol) and THF/H_2O (1:1, 10 mL) was treated with $LiOH{\cdot}H_2O$ (34 mg, 0.822 mmol, 2.1 equiv) at ambient temperature for 4 h. Solvent volume was reduced by half under reduced pressure, diluted to 10 mL with H_2O and acidified to pH 1 (using 1M HCl). The aqueous phase was transferred to a separatory funnel and extracted into CH_2Cl_2 (3 × 20 mL). Organic extracts were combined, dried, filtered and solvent removed under reduced pressure to afford the title compound as a yellow solid (132.3 mg, 79%).

^{1}H NMR (DMSO-d_6, 400 MHz): δ 10.67 (s, 1H) 8.99 (d, *J* = 1.2 1H), 8.87 (d, *J* = 1.2 1H), 8.41–8.37 (m, 2H), 8.07 (d, *J* = 8.4, 2H), 7.82 (t, *J* = 7.9, 1H), 7.12 (d, *J* = 8.4, 2H), 4.04 (t, *J* = 7.1, 2H), 3.87 (s, 3H), 2.22 (t, *J* = 7.1, 2H), 1.76–1.52 (m, 4H), 1.39–1.33 (m, 2H).

^{13}C NMR (DMSO-d_6, 100 MHz): δ 174.5, 165.4, 163.5, 163.3, 162.3, 138.3, 133.9, 132.1, 129.9, 129.1, 127.6, 126.3, 125.1, 124.2, 122.6, 122.0, 121.9, 113.8, 55.5, 39.5*, 33.5, 27.3, 26.1, 24.3.

HRMS (ESI) for $C_{26}H_{24}N_2O_6$ [M + H$^+$] calcd, 461.1707; found, 461.1700

*Determined by HSQC

***N*-(2-(6-(Hydroxyamino)-6-oxohexyl)-1,3-dioxo-2,3-dihydro-1*H*-benzo[*de*]isoquinolin-5-yl)-4-methoxybenzamide (KNH020)**

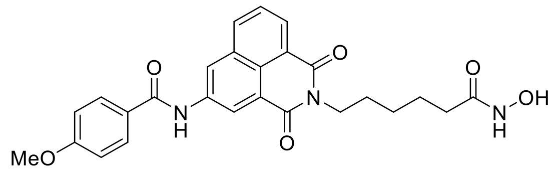

A stirring solution of carboxylic acid **15** (81 mg, 0.175 mmol) and THF (3 mL) was charged with Et_3N (46 µL, 0.330 mmol, 1.9 equiv) and $ClCO_2Et$ (18 µL, 0.189 mmol, 1.1 equiv) and stirred at ambient temperature for 1 h. The solution was filtered to remove $Et_3N{\cdot}HCl$ and the filtrate was directly added to a freshly prepared solution of NH_2OH in MeOH (0.5 M, 3 mL) and allowed to stir for 24 h. Excess solvent was removed and the crude residue was triturated with MeCN to provide the title compound as a yellow solid (61 mg, 73%).

^{1}H NMR (DMSO-d_6, 400 MHz): δ 10.68 (s, 1H), 10.33 (s, 1H), 8.99 (s, 1H). 8.87 (s, 1H), 8.66 (s, 1H), 8.41–8.37 (m, 2H), 8.07 (d, *J* = 8.6, 2H), 7.83 (t, *J* = 7.8, 1H), 7.12 (d, *J* = 8.6, 2H), 4.04 (t, *J* = 7.1, 2H), 3.87 (s, 3H), 1.96 (t, *J* = 7.1, 2H), 1.67–1.52 (m, 4H), 1.36–1.30 (m, 2H).

^{13}C NMR (DMSO-d_6, 100 MHz): δ 169.1, 165.4, 163.5, 163.3, 162.3, 138.3, 133.9, 132.1, 129.9, 129.1, 127.6, 126.3, 125.1, 124.2, 122.6, 122.0, 121.9, 113.8, 55.5, 39.5, 32.2, 27.4, 26.2, 24.9.

HRMS (ESI) for $C_{26}H_{25}N_3O_6$ [M + H$^+$] calcd, 476.1816; found, 476.1823.

Methyl 6-(1,3-dioxo-5-propionamido-1*H*-benzo[de]isoquinolin-2(3*H*)-yl)hexanoate (16)

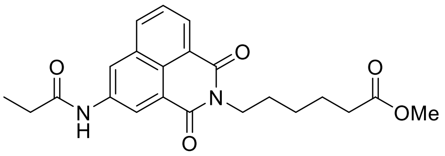

Using the general procedure, 3-bromo-1,8-naphthalimide **11** (192 mg, 0.475 mmol), propionamide (67 mg, 0.922 mmol, 1.9 equiv), G3-Xantphos (4.7 mg, 0.005 mmol, 0.01 equiv), Cs_2CO_3 (225 mg, 0.691 mmol, 1.46 equiv) in 1,4-dioxane (5.0 mL) were allowed to react at 100 °C for 70 min. The crude material was dissolved in MeOH, loaded onto SiO_2 and purified using column chromatography (1:1 EtOAc/pet. spirits, R_f = 0.33) to provide the desired compound in >95% purity as a white solid (111.8 mg, 59%).

^{1}H NMR (DMSO-d_6, 500 MHz): δ 10.48 (s, 1H), 8.78 (d, J = 1.8, 1H), 8.59 (d, J = 1.8, 1H), 8.34 (d, J = 7.8, 2H), 7.79 (t, J = 7.8, 1H), 4.03–4.00 (m, 2H), 3.56 (s, 3H), 2.43 (q, J = 7.5, 2H), 2.31 (t, J = 7.4, 2H), 1.65–1.54 (m, 4H), 1.37–1.31 (m, 2H), 1.14 (t, J = 7.5, 3H).

^{13}C NMR (DMSO-d_6, 125 MHz): δ 173.4, 172.8, 163.5, 163.3, 138.1, 133.7, 132.2, 128.9, 127.6, 123.92, 123.88, 122.7, 121.9, 120.6, 51.2, 39.8*, 33.1, 29.7, 27.2, 26.0, 24.2, 9.6.

HRMS (ESI) for $C_{22}H_{24}N_2O_5$ [M + H$^+$] calcd, 397.1758; found, 397.1752.

*Determined using HSQC

6-(1,3-Dioxo-5-propionamido-1*H*-benzo[*de*]isoquinolin-2(3*H*)-yl)hexanoic acid (17)

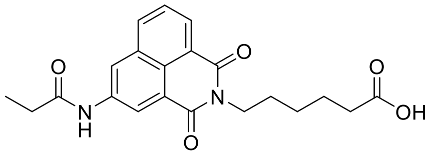

A stirring solution of methyl ester **16** (176 mg, 0.443 mmol) and THF/H_2O (1:1, 10 mL) was charged with LiOH·H_2O (34 mg, 0.810 mmol, 1.8 equiv) and stirred at ambient temperature for 24 h. Solvent volume was reduced by half *in vacuo*, diluted with H_2O (10 mL) and acidified to pH 1 with 1 M HCl. The aqueous residue was transferred to a separatory funnel and extracted into 10% MeOH/CH_2Cl_2 (3 × 20 mL). Organic extracts were combined, dried, filtered and solvent removed under reduced pressure to afford the title compound as a yellow solid (168 mg, 96%).

^{1}H NMR (DMSO-d_6, 500 MHz): δ 10.49 (s, 1H), 8.80 (d, J = 1.8, 1H), 8.60 (d, J = 1.8, 1H), 8.35 (d, J = 7.7, 2H), 7.80 (t, J = 7.7, 1H), 4.02 (t, J = 7.3, 2H), 2.43 (q, J = 7.4, 2H), 2.20 (t, J = 7.3, 2H), 1.64–1.53 (m, 4H), 1.37–1.31 (m, 2H), 1.14 (t, J = 7.4, 3H).

^{13}C NMR (DMSO-d_6, 125 MHz): δ 174.5, 172.8, 163.5, 163.2, 138.1, 133.7, 132.1, 128.9, 127.6, 123.9, 123.8, 122.7, 121.9, 120.6, 39.9*, 33.7, 29.6, 27.3, 26.1, 24.3, 9.5.

HRMS (ESI) for $C_{21}H_{22}N_2O_5$ [M + H$^+$] calcd, 383.1601; found, 383.1606.

*Determined using HSQC

6-(1,3-Dioxo-5-propionamido-1*H*-benzo[*de*]isoquinolin-2(3*H*)-yl)-*N*-hydroxyhexanamide (KNH021)

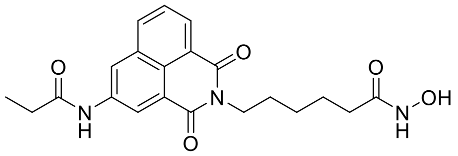

A stirring solution of carboxylic acid **17** (44 mg, 0.115 mmol) and THF (2 mL) was charged with Et_3N (30 µL, 0.215 mmol, 1.87 equiv) and $ClCO_2Et$ (12 µL, 0.126 mmol, 1.10 equiv) before stirring for 20 min at ambient temperature. After this time, the solution was filtered to remove $Et_3N{\cdot}HCl$ and the filtrate was added to a freshly prepared solution of NH_2OH in MeOH (0.5 M, 2 mL) at room temperature. After 21 h, the solvent was removed under reduced pressure and the crude residue was dissolved in H_2O (5 mL) and transferred to a separatory funnel. The aqueous phase was extracted with EtOAc (4 × 5 mL), organic phases were combined, washed with 1 M HCl (5 mL), brine (5 mL), dried, filtered and solvent removed under reduced pressure to provide a yellow solid. This crude material was recrystallised using EtOH to provide the title compound as a yellow solid (41.0 mg, 90%).

^{1}H NMR (DMSO-d_6, 400 MHz): δ 10.49 (s, 1H), 10.33 (s, 1H), 8.80 (s, 1H), 8.66 (s, 1H), 8.61 (s, 1H), 8.36 (d, *J* = 7.9, 2H), 7.80 (t, *J* = 7.6, 1H), 4.02 (t, *J* = 7.0, 2H), 2.43 (q, *J* = 7.6, 2H), 1.95 (t, *J* = 7.2, 2H), 1.66–1.50 (m, 4H), 1.35–1.29 (m, 2H), 1.14 (t, *J* = 7.6, 3H).

^{13}C NMR (DMSO-d_6, 125 MHz): δ 172.9, 163.5, 163.3, 138.2, 133.7, 132.2, 129.0, 127.7, 124.0, 123.9, 122.7, 121.9, 120.7, 39.4*, 31.8, 29.3, 27.0, 25.9, 24.6. 9.2

HRMS (ESI) for $C_{21}H_{23}N_3O_5$ [M + H$^+$] calcd, 398.1710; found 398.1718

*Determined using HSQC

S1.4 References

(1) Hearn, K. N.; Nalder, T. D.; Cox, R. P.; Maynard, H. D.; Bell, T. D. M.; Pfeffer, F. M.; Ashton, T. D. Modular Synthesis of 4-Aminocarbonyl Substituted 1,8-Naphthalimides and Application in Single Molecule Fluorescence Detection. *Chem. Commun.* **2017**, *53* (91), 12298–12301. https://doi.org/10.1039/C7CC07922B.

(2) Moseley, J. D.; Moss, W. O.; Welham, M. J.; Ancell, C. L.; Banister, J.; Bowden, S. A.; Norton, G.; Young, M. J. A New Approach to Rapid Parallel Development of Four Neurokinin Antagonists. Part 2. Synthesis of ZD6021 Cyano Acid. *Org. Process Res. Dev.* **2003**, *7* (1), 58–66. https://doi.org/10.1021/op020065h.

S1.5 NMR Spectra

Figure S1 – ^{1}H and ^{13}C NMR of **5**

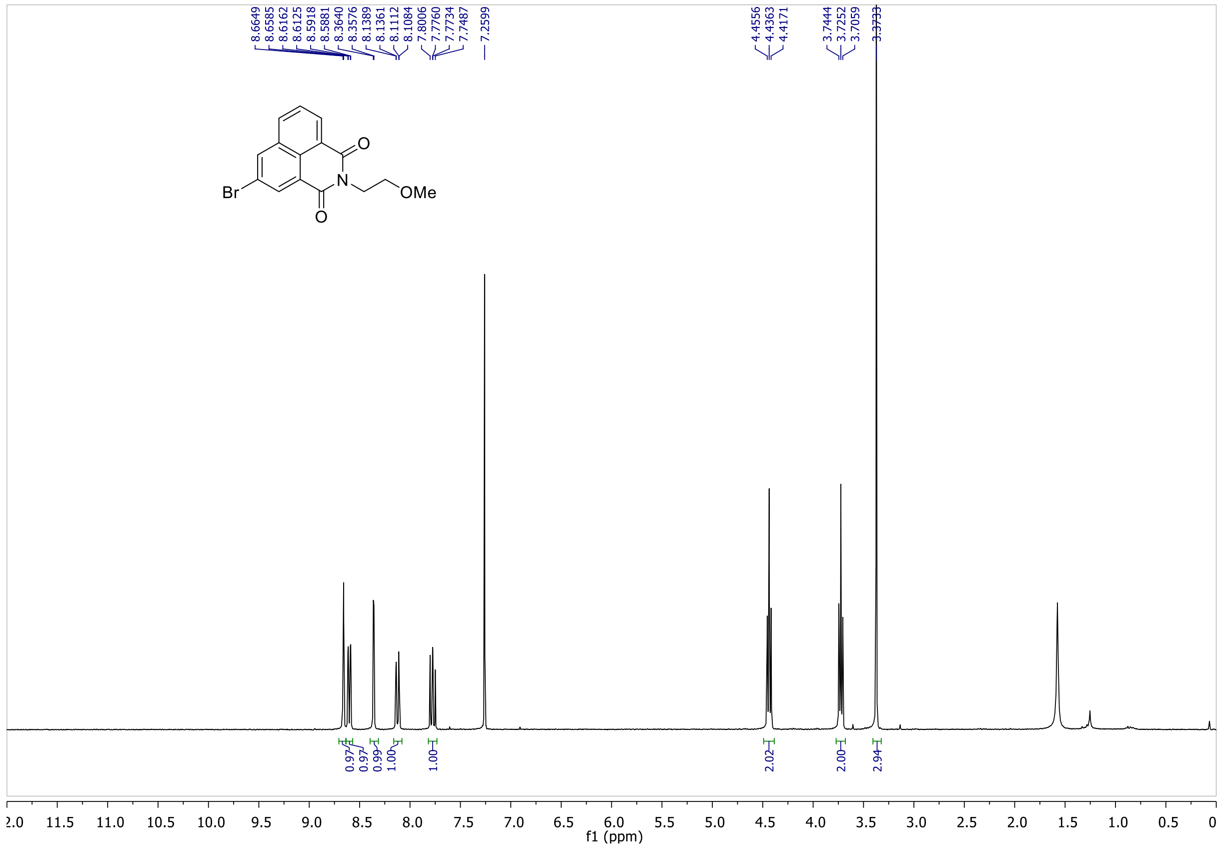

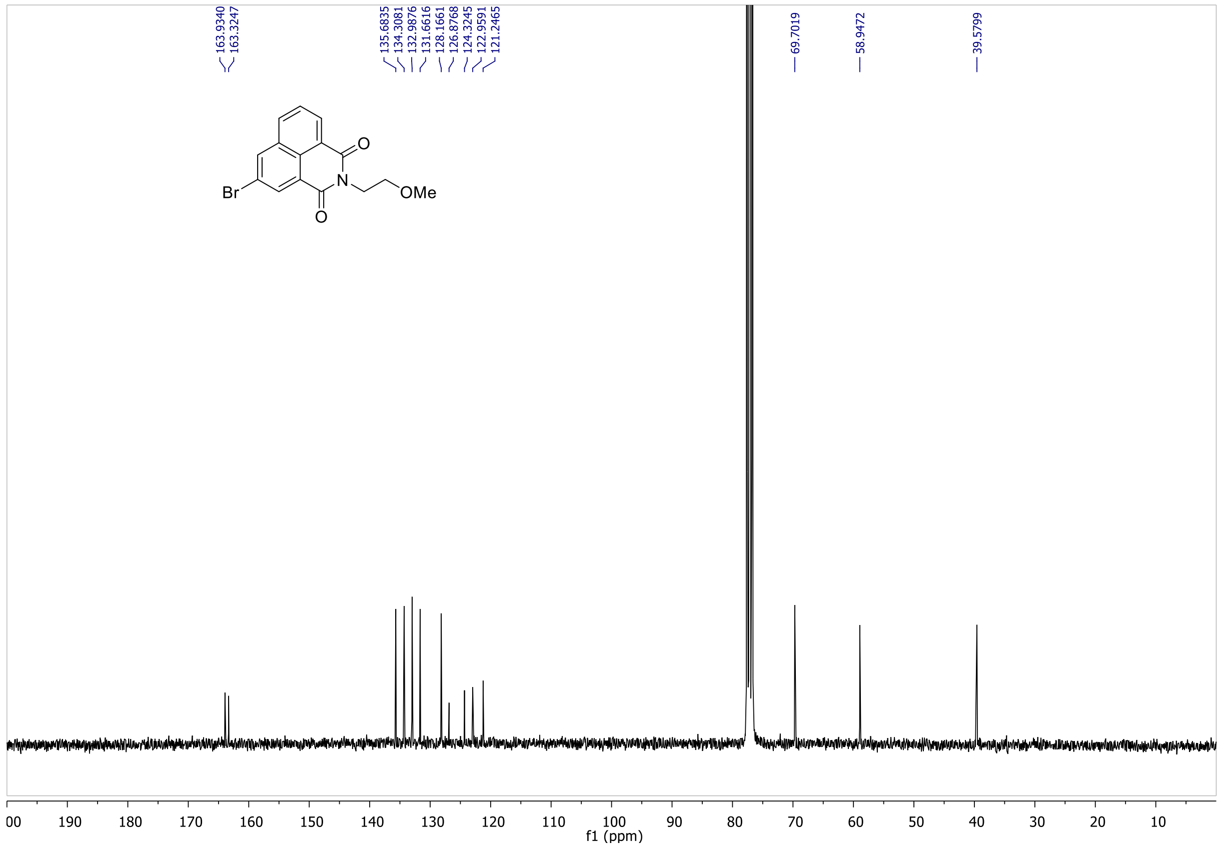

Figure S2 – ^{1}H and ^{13}C NMR of **6**

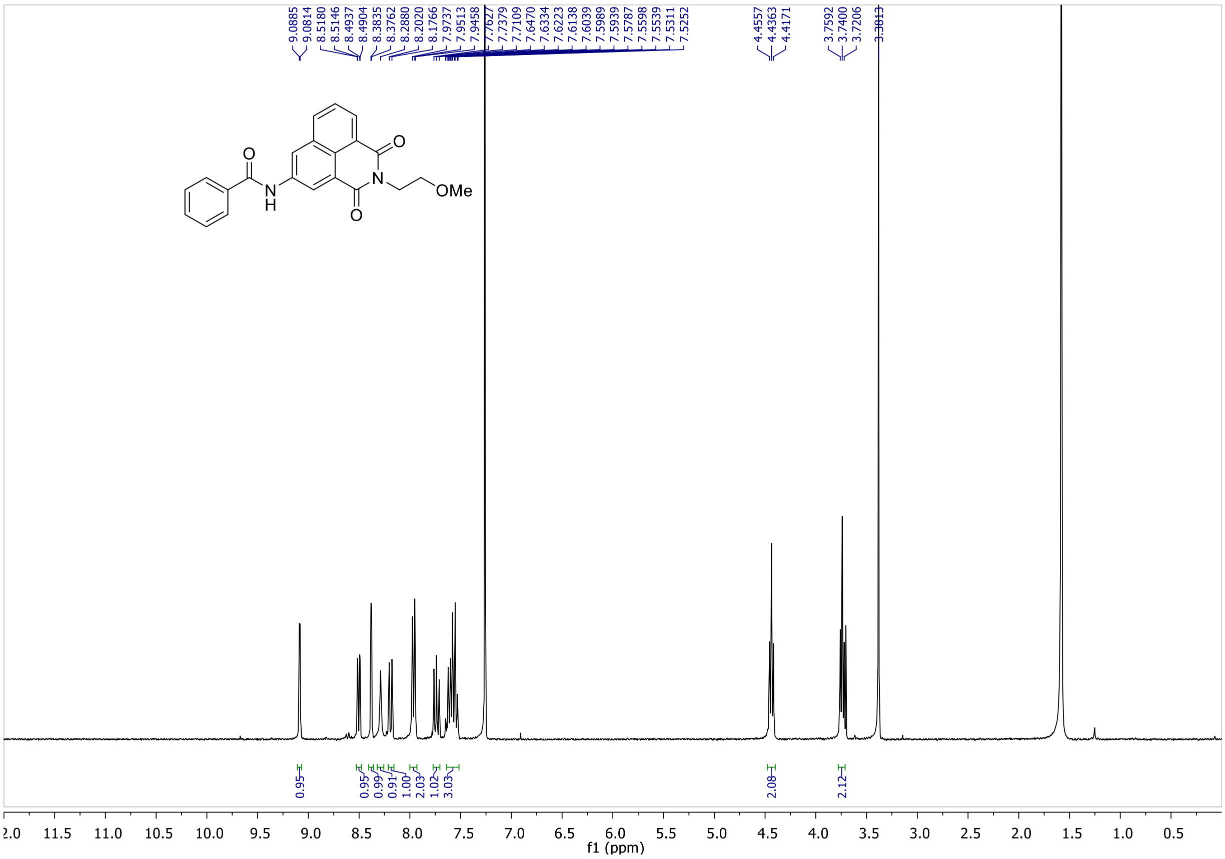

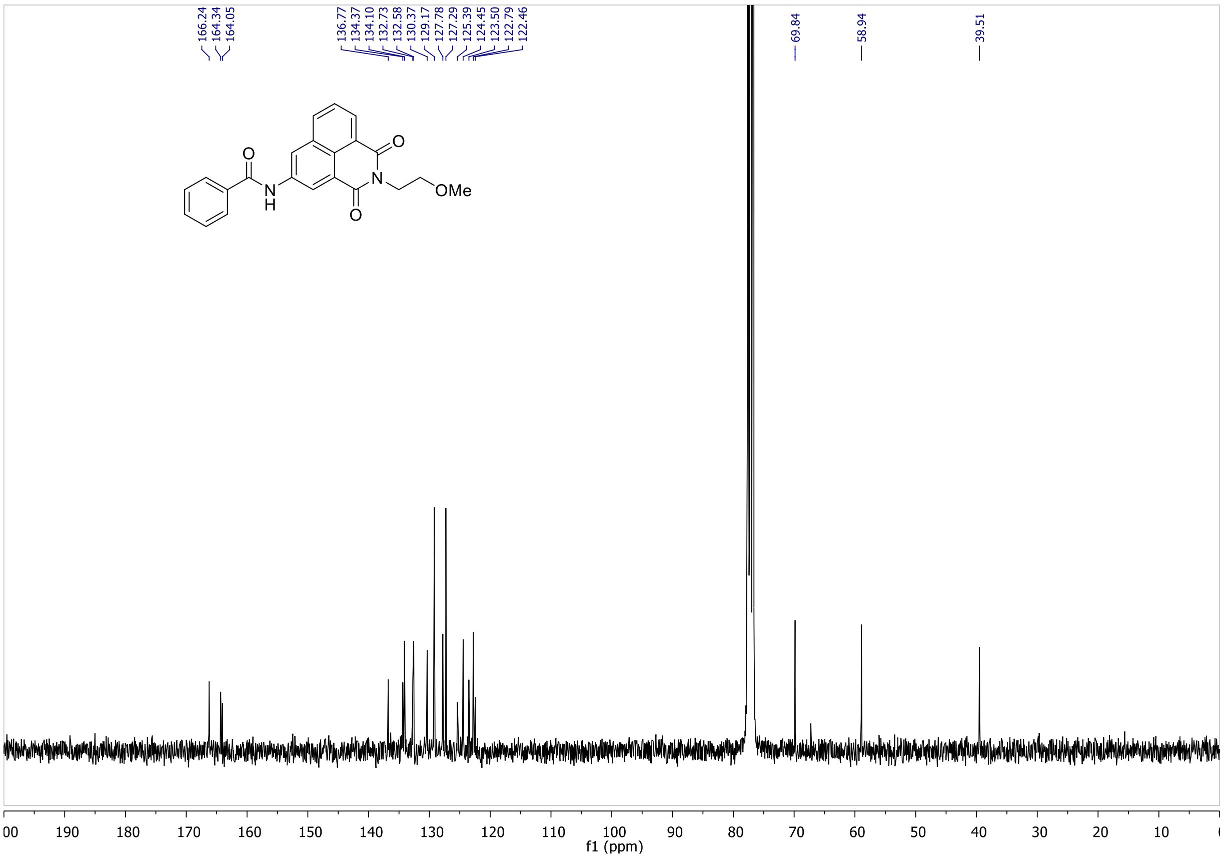

Figure S3 – ^{1}H and ^{13}C NMR of **7**

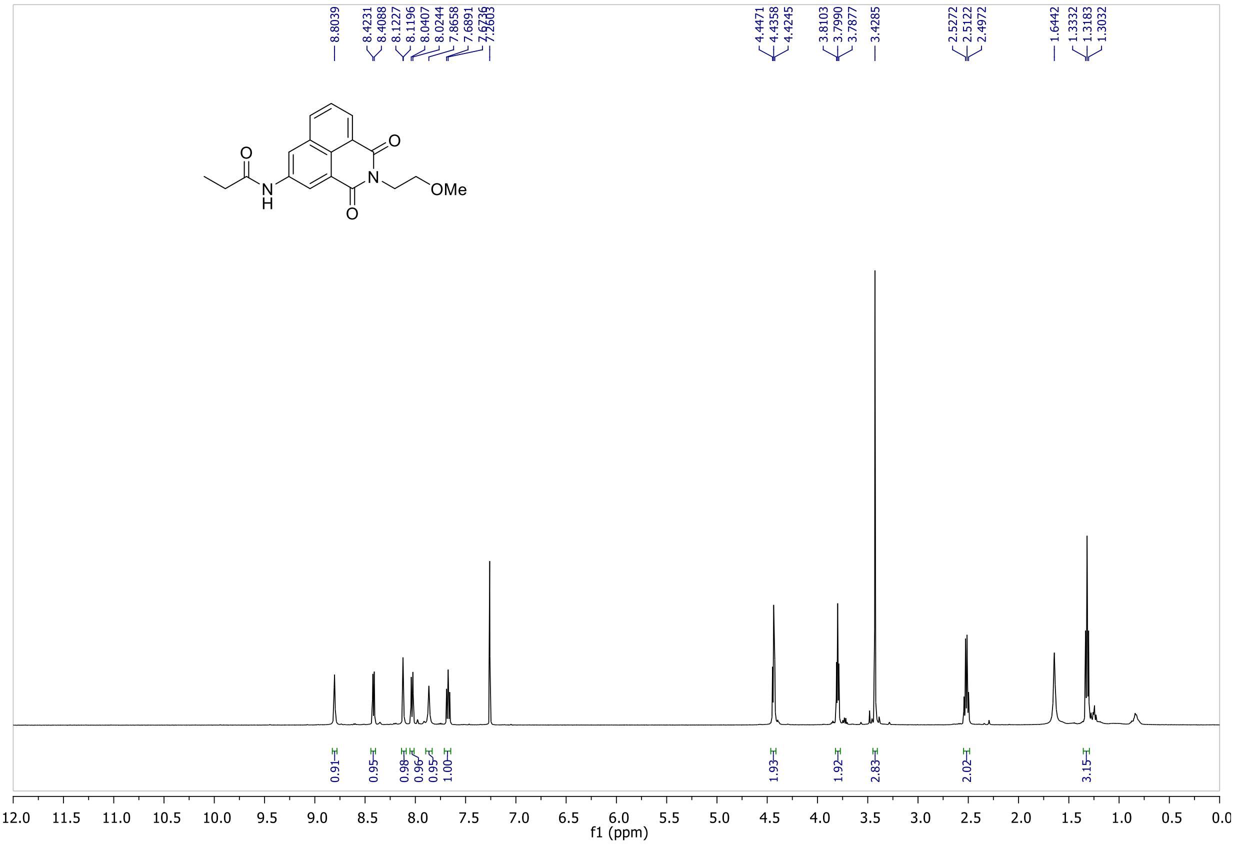

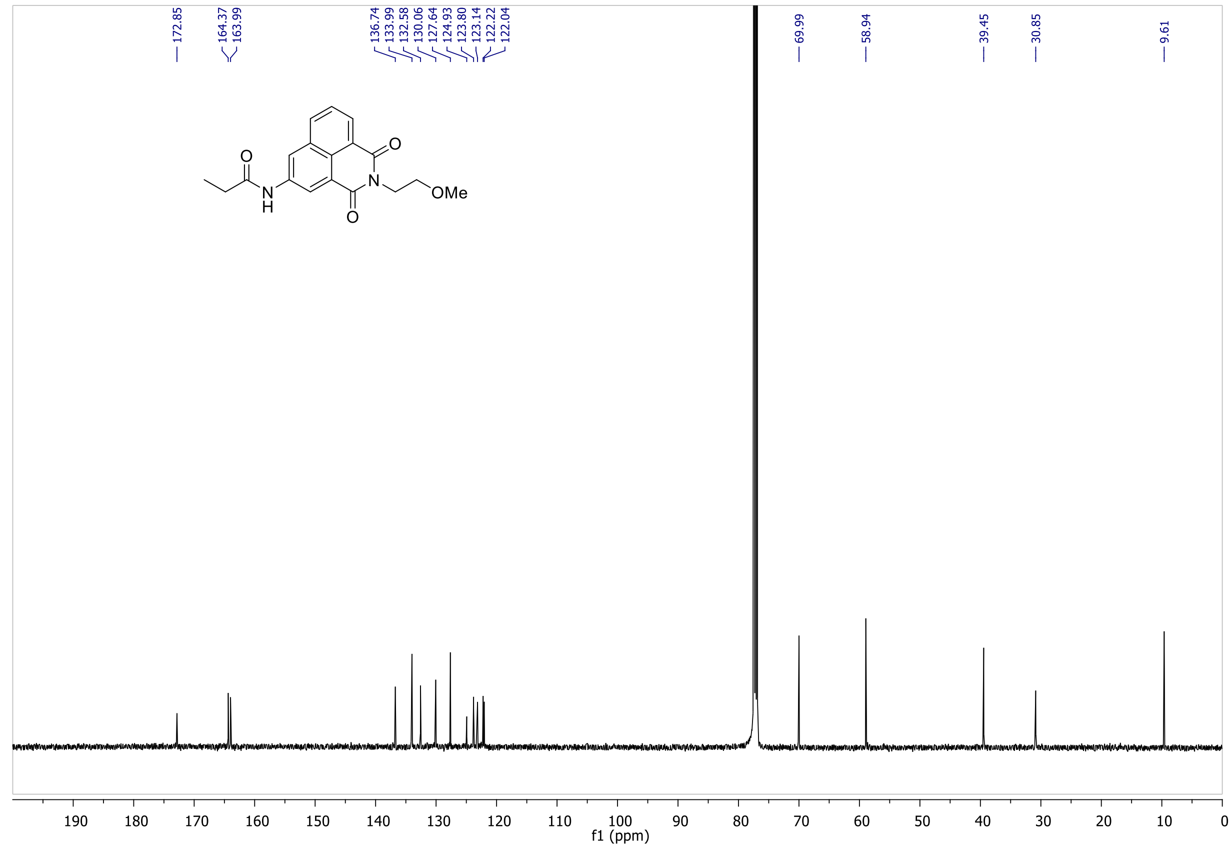

Figure S4 – ^{1}H and ^{13}C NMR of **8**

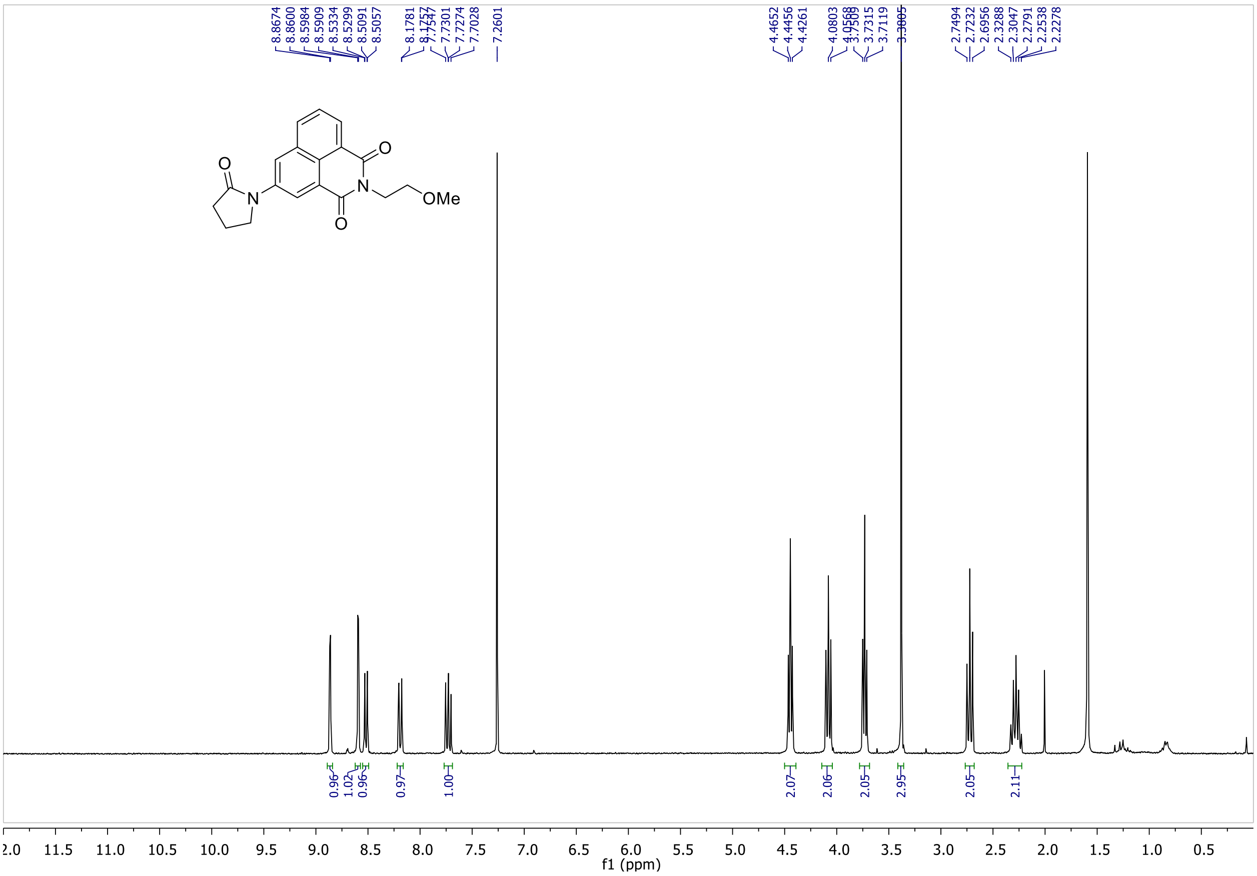

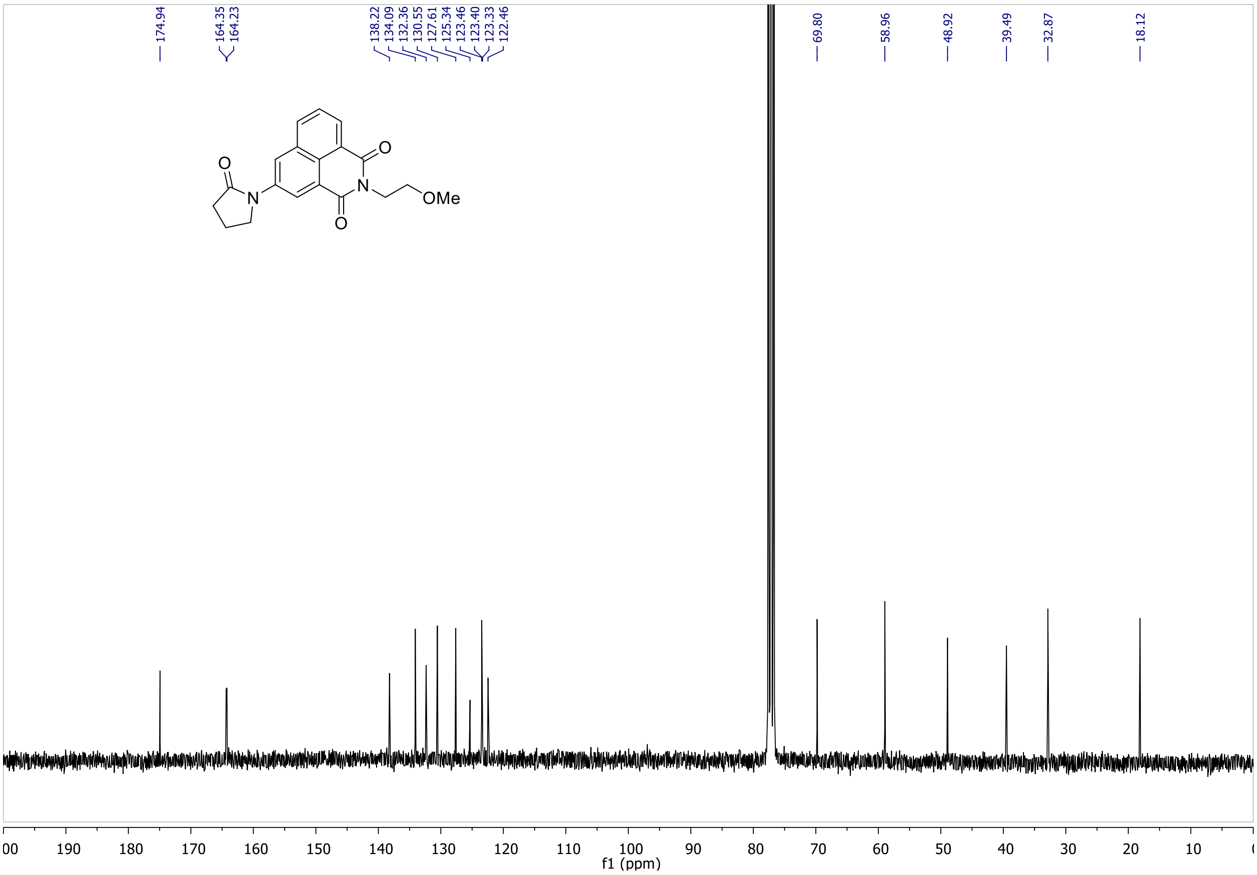

Figure S5 – ^{1}H and ^{13}C NMR of **9**

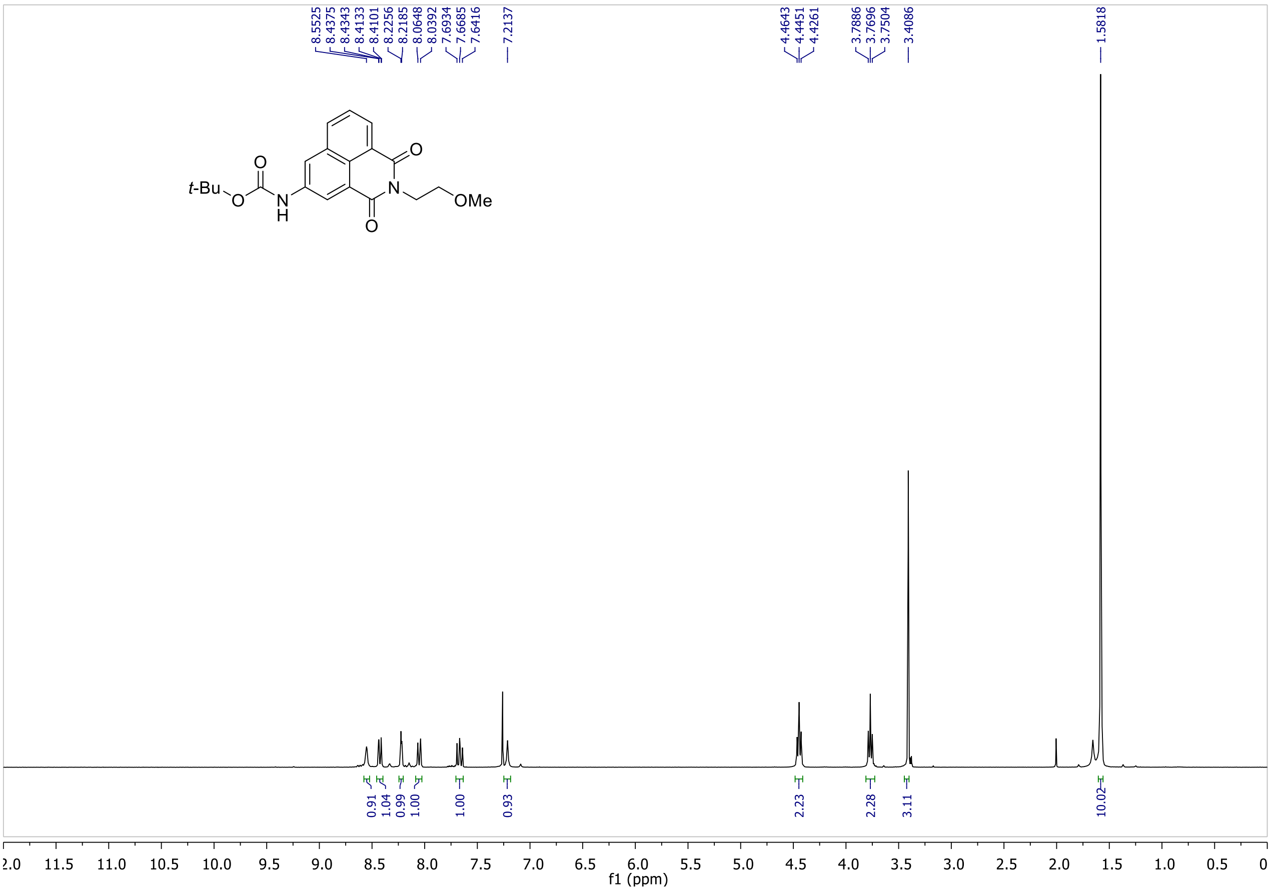

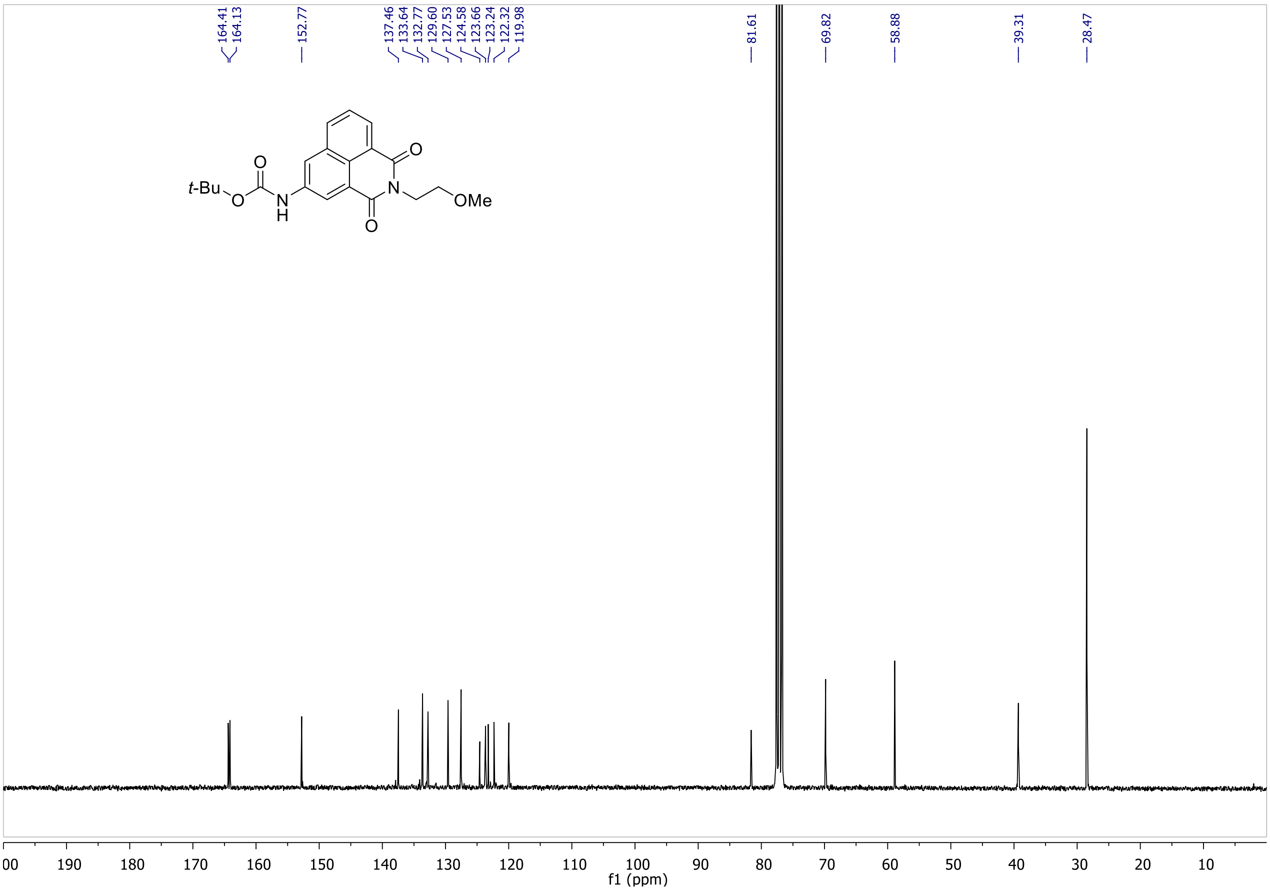

Figure S6 – ^{1}H and ^{13}C NMR of **10**

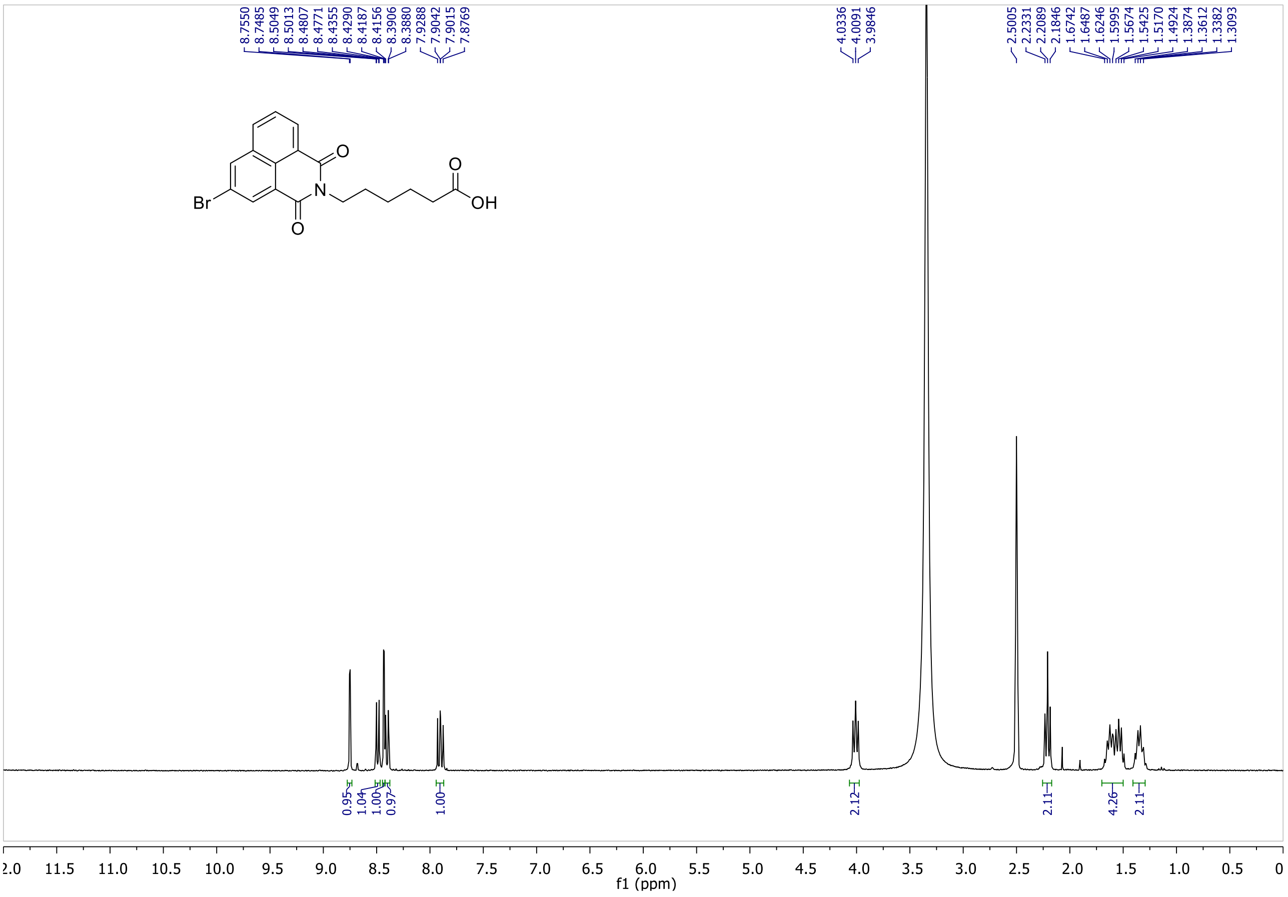

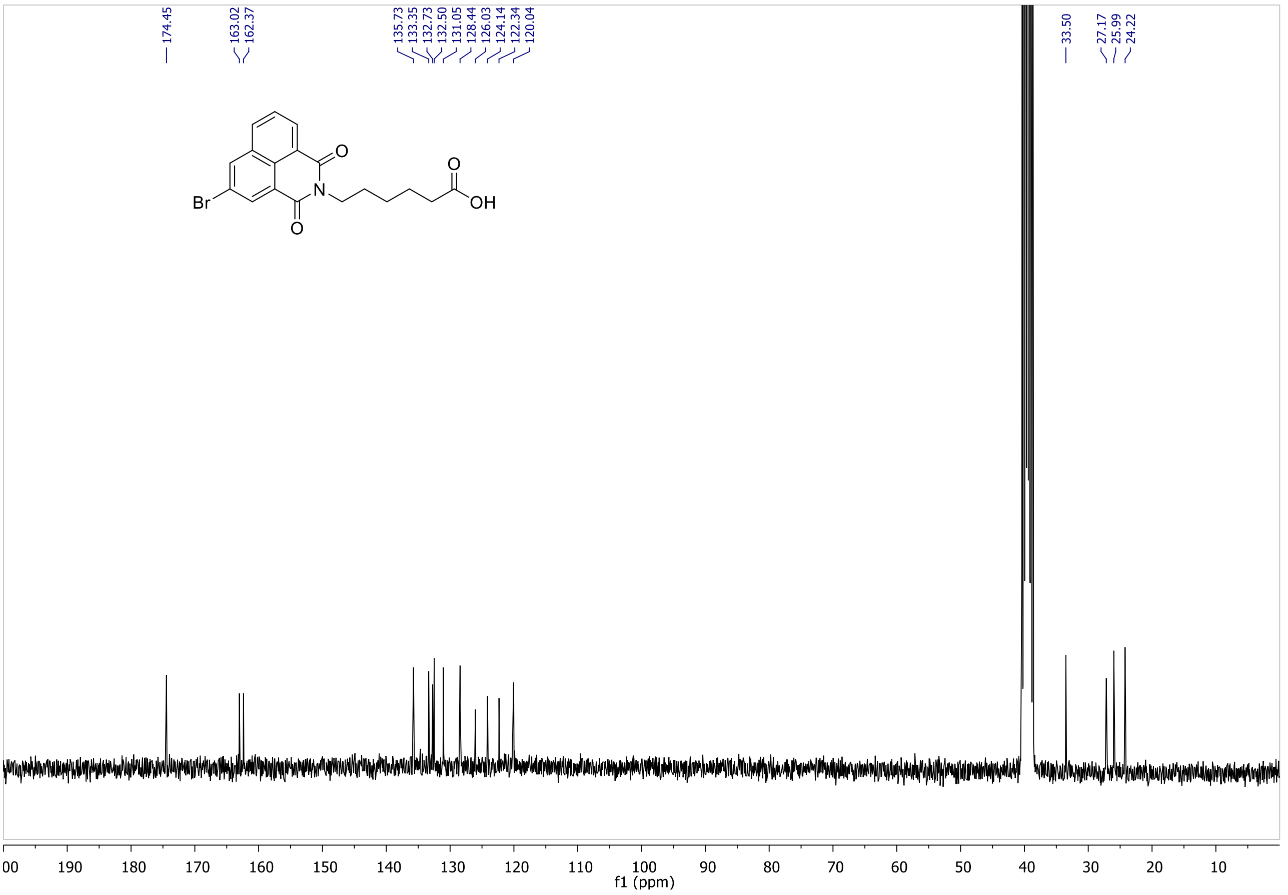

Figure S7 – ^{1}H and ^{13}C NMR of **11**

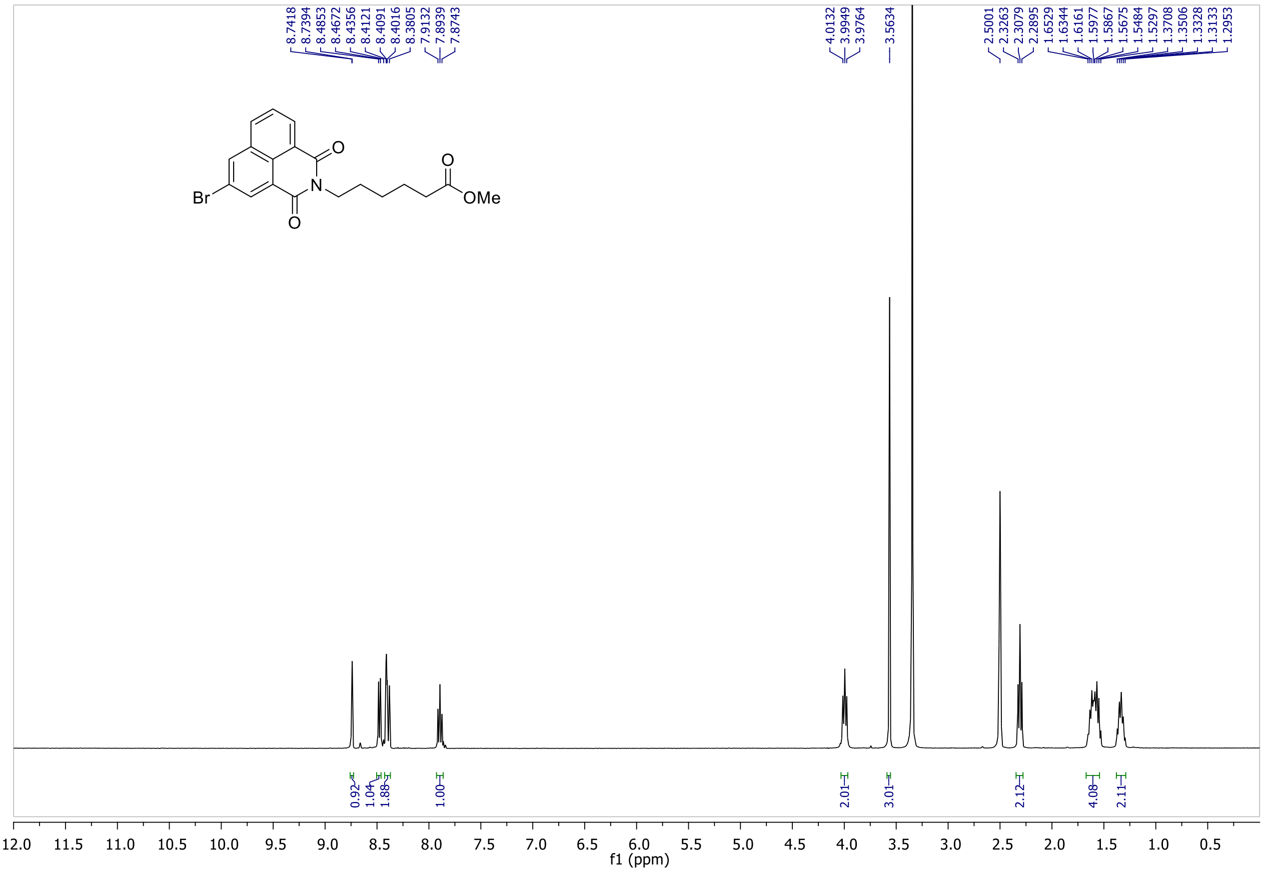

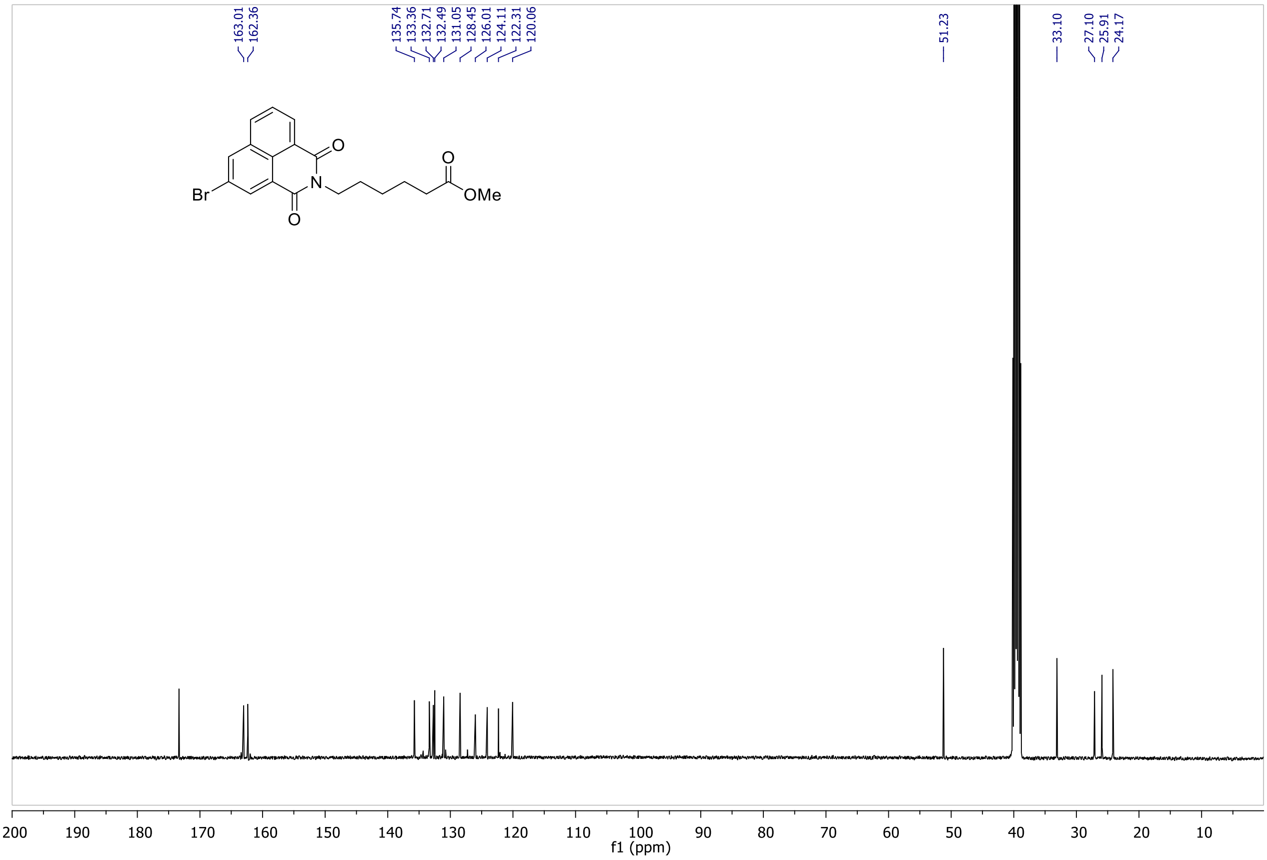

Figure S8 – ^{1}H and ^{13}C NMR of **12**

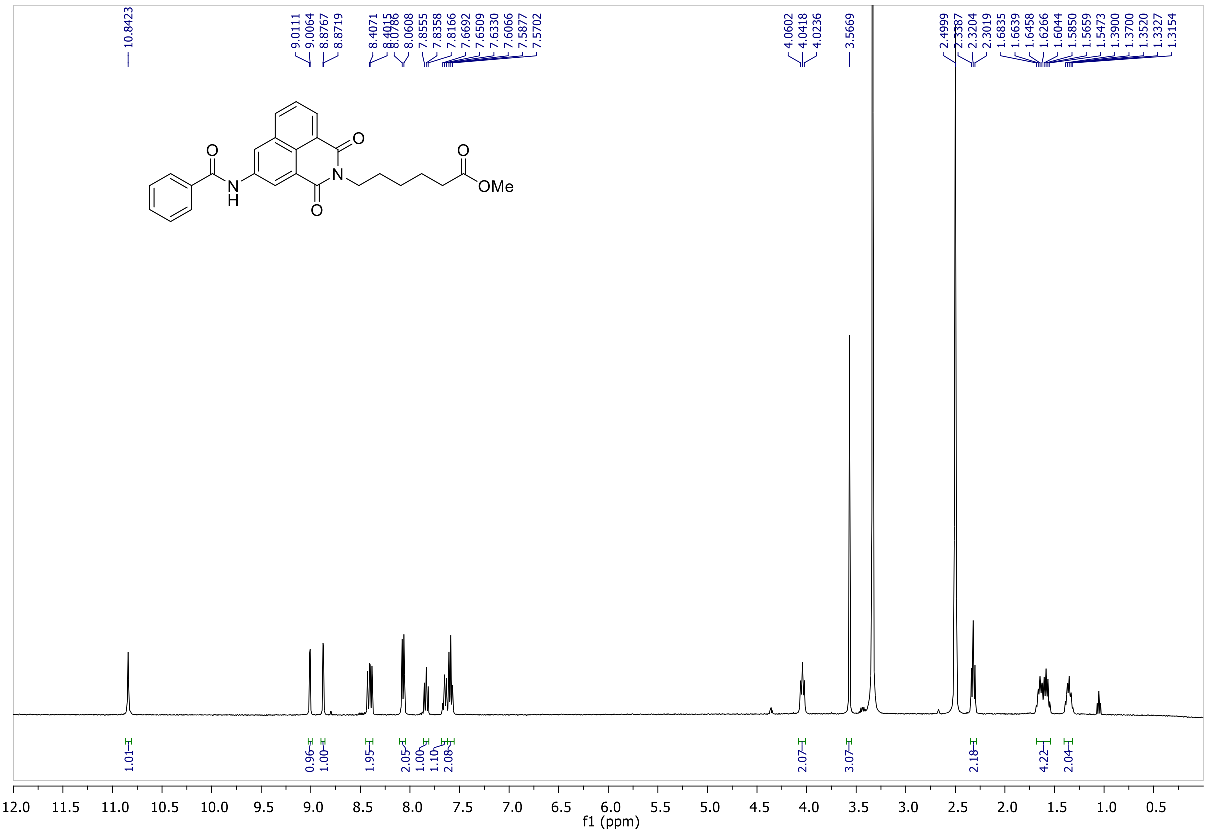

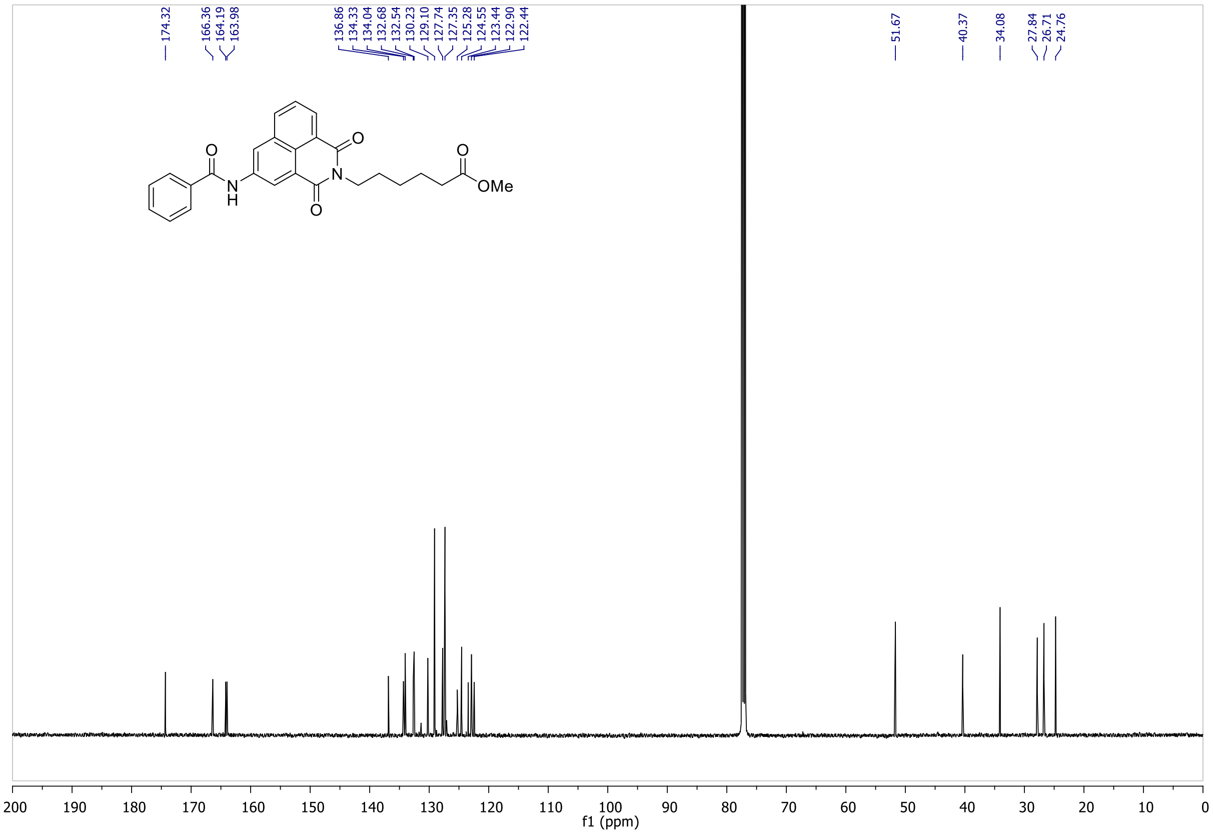

Figure S9 – ^{1}H and ^{13}C NMR of **13**

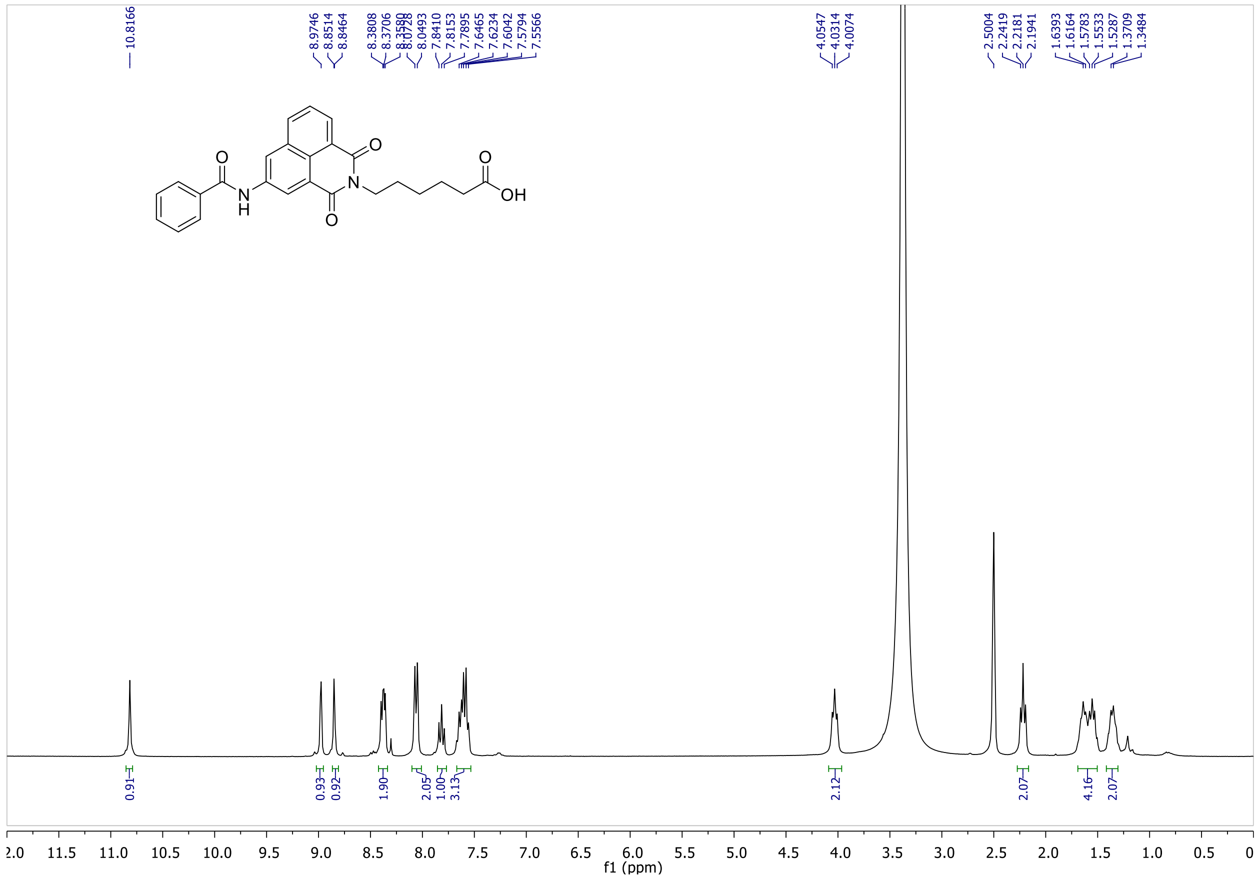

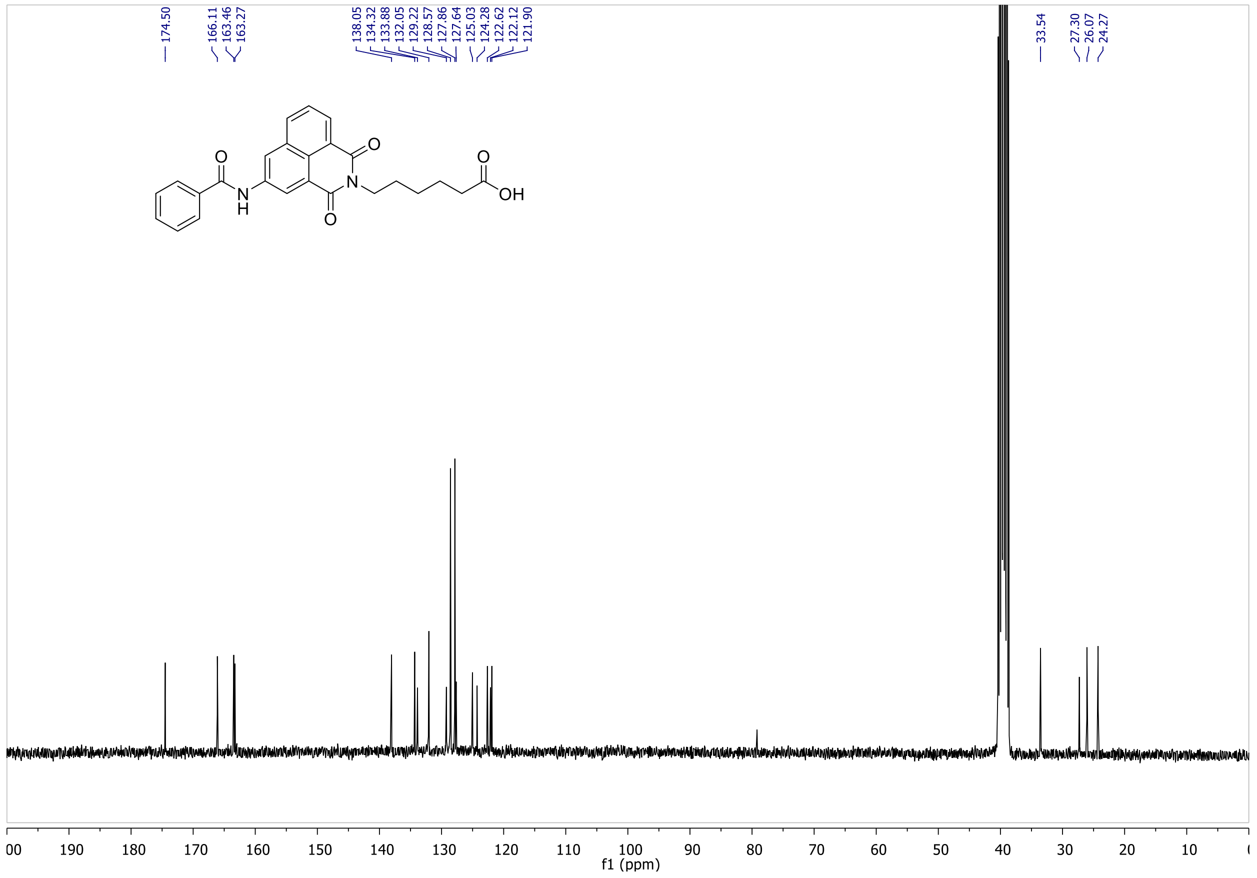

Figure S10 – ^{1}H and ^{13}C NMR of **KNH019**

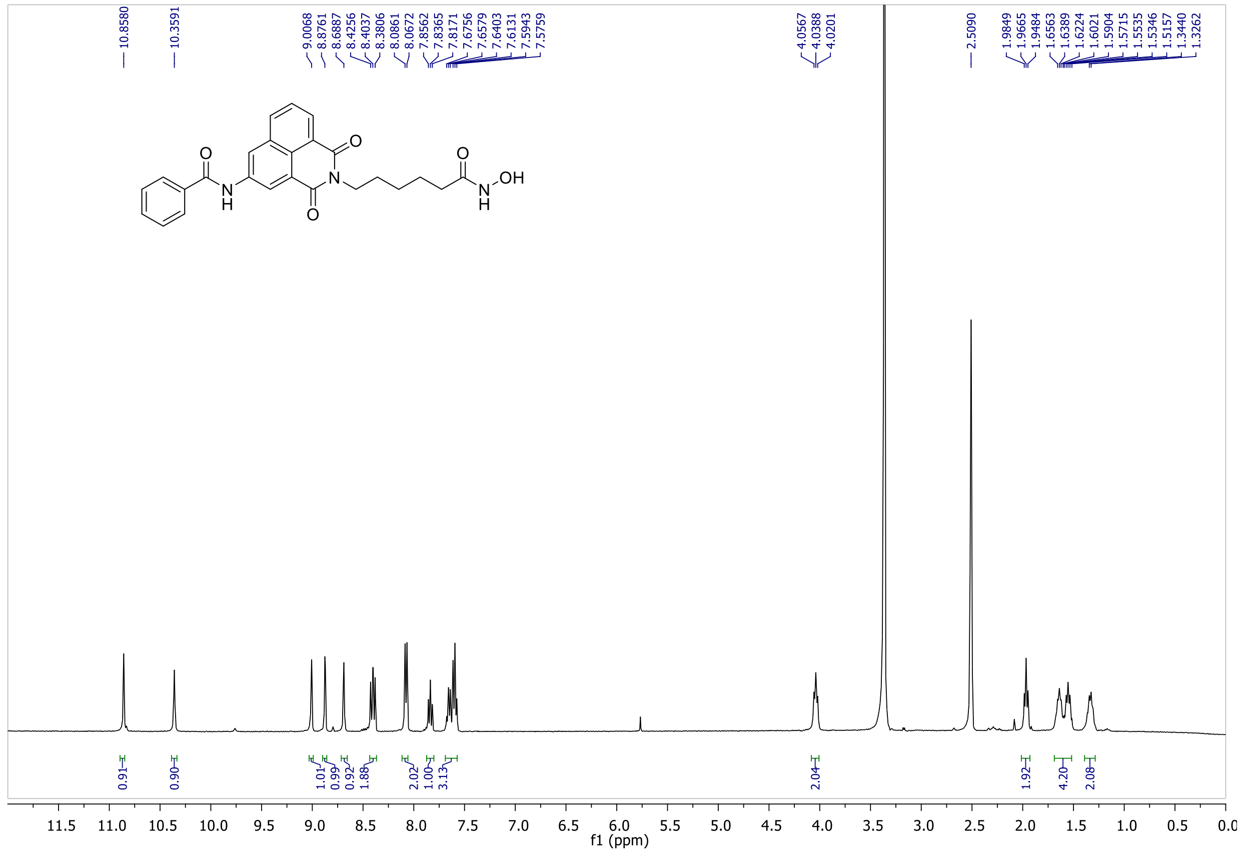

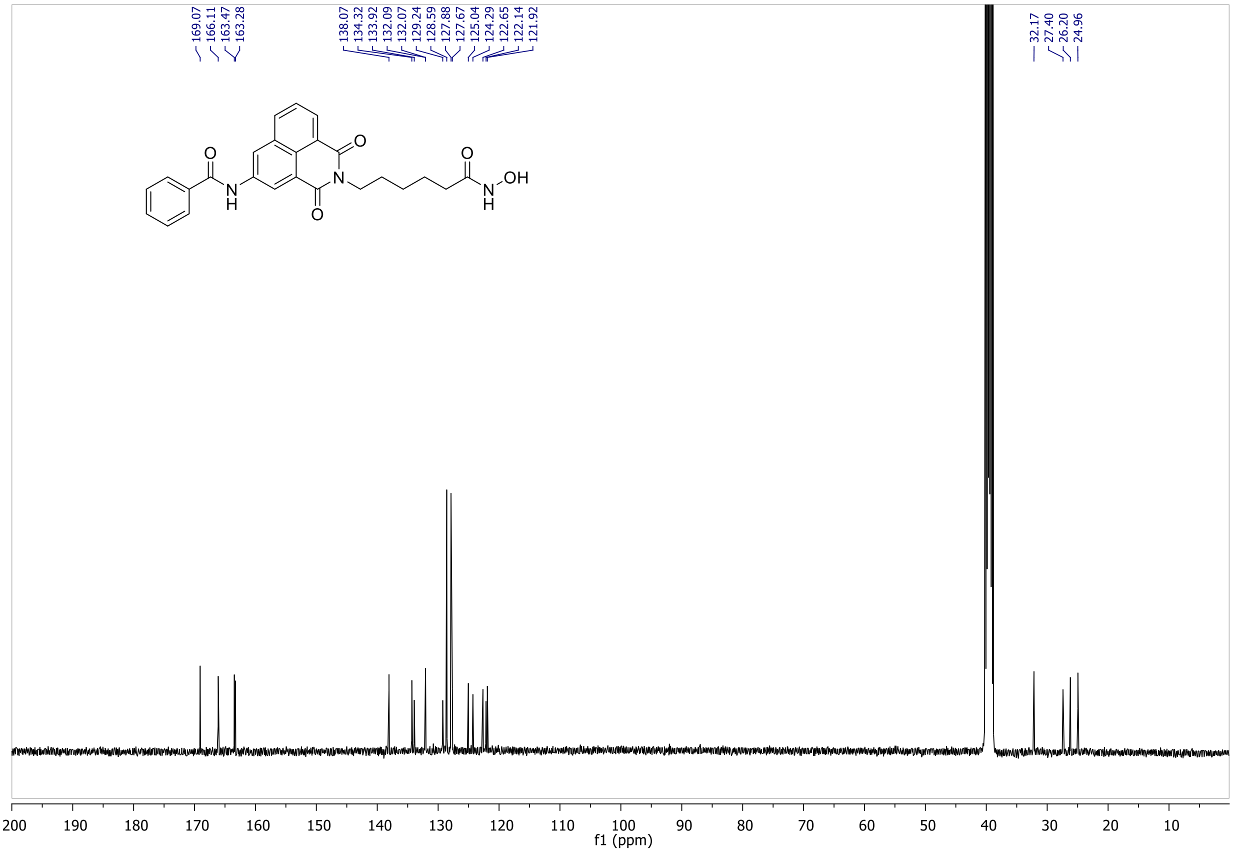

Figure S11 – ^{1}H and ^{13}C NMR of **14**

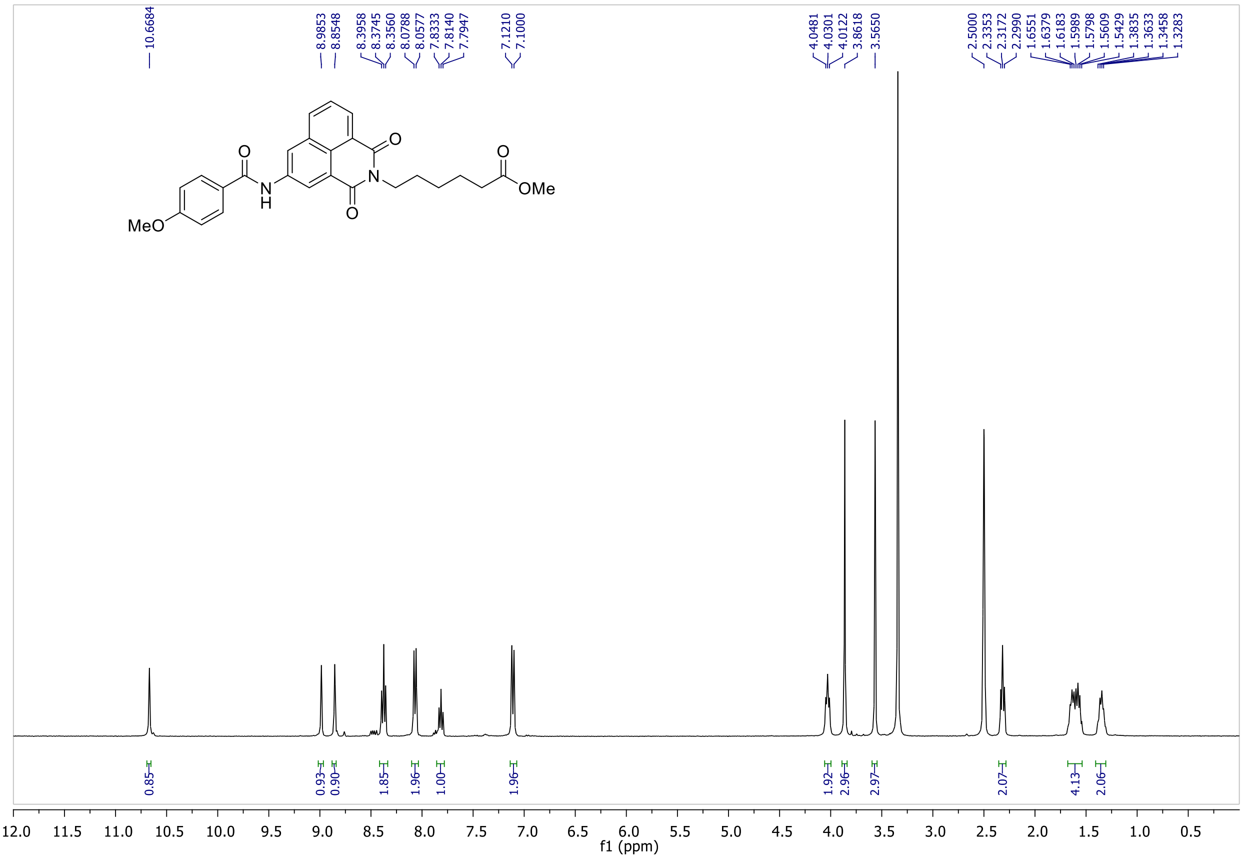

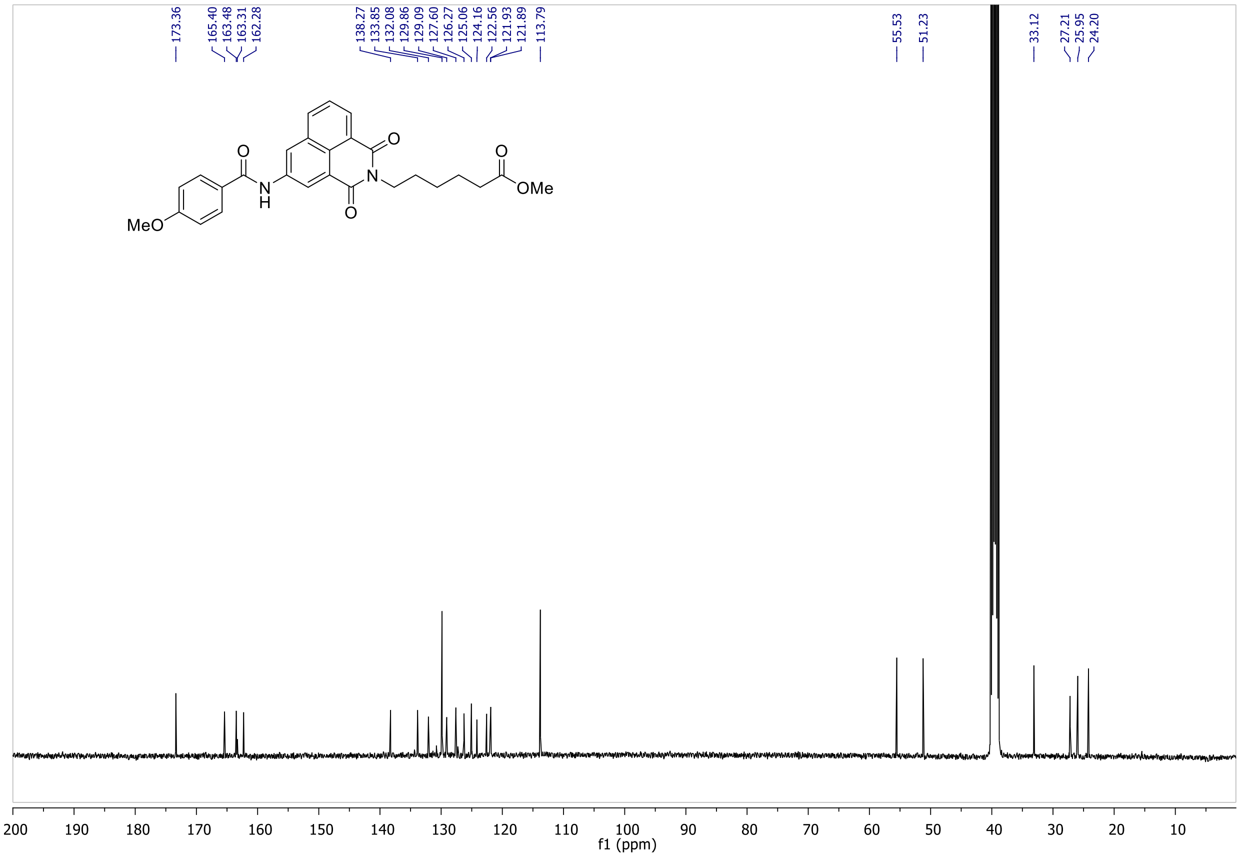

Figure S12 – ^{1}H and ^{13}C NMR of **15**

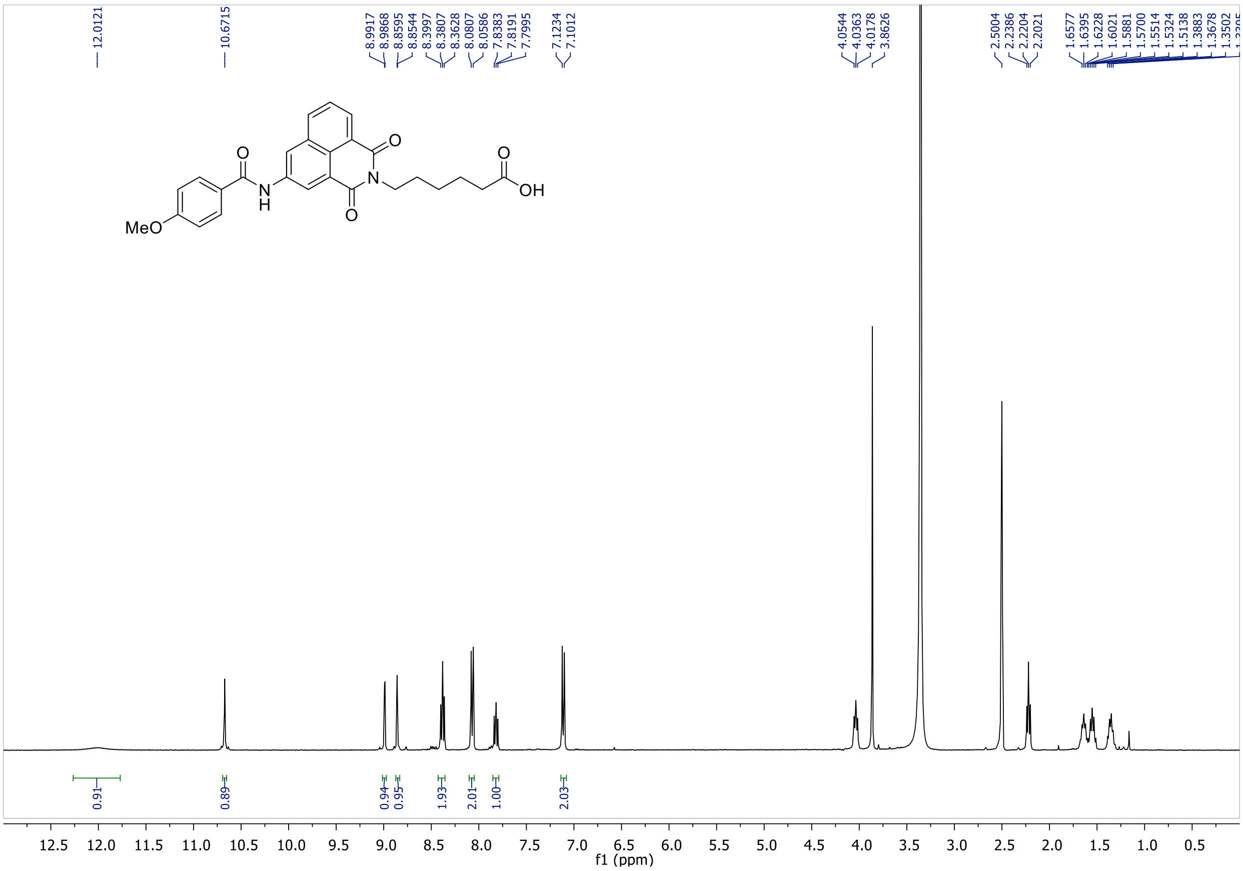

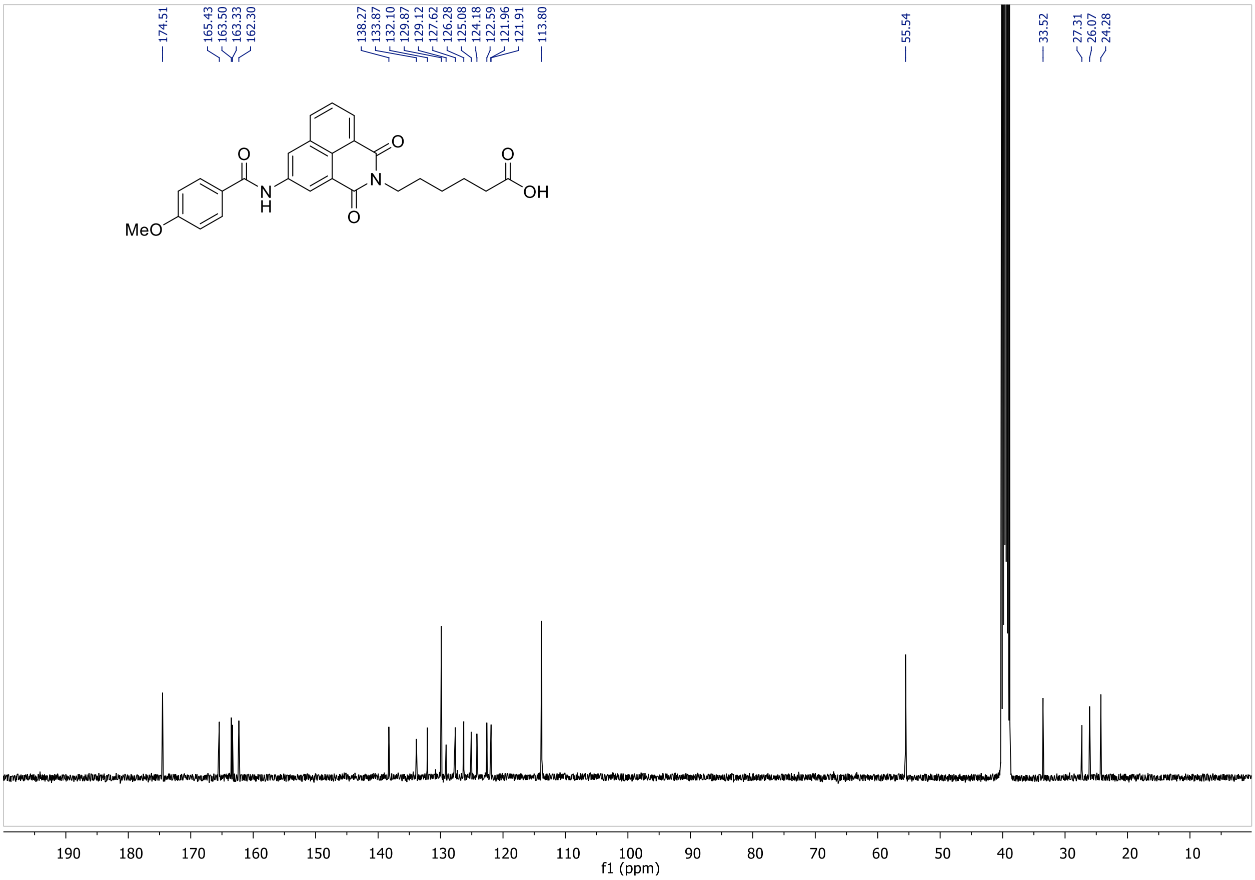

Figure S13 – ^{1}H and ^{13}C NMR of **KNH020**

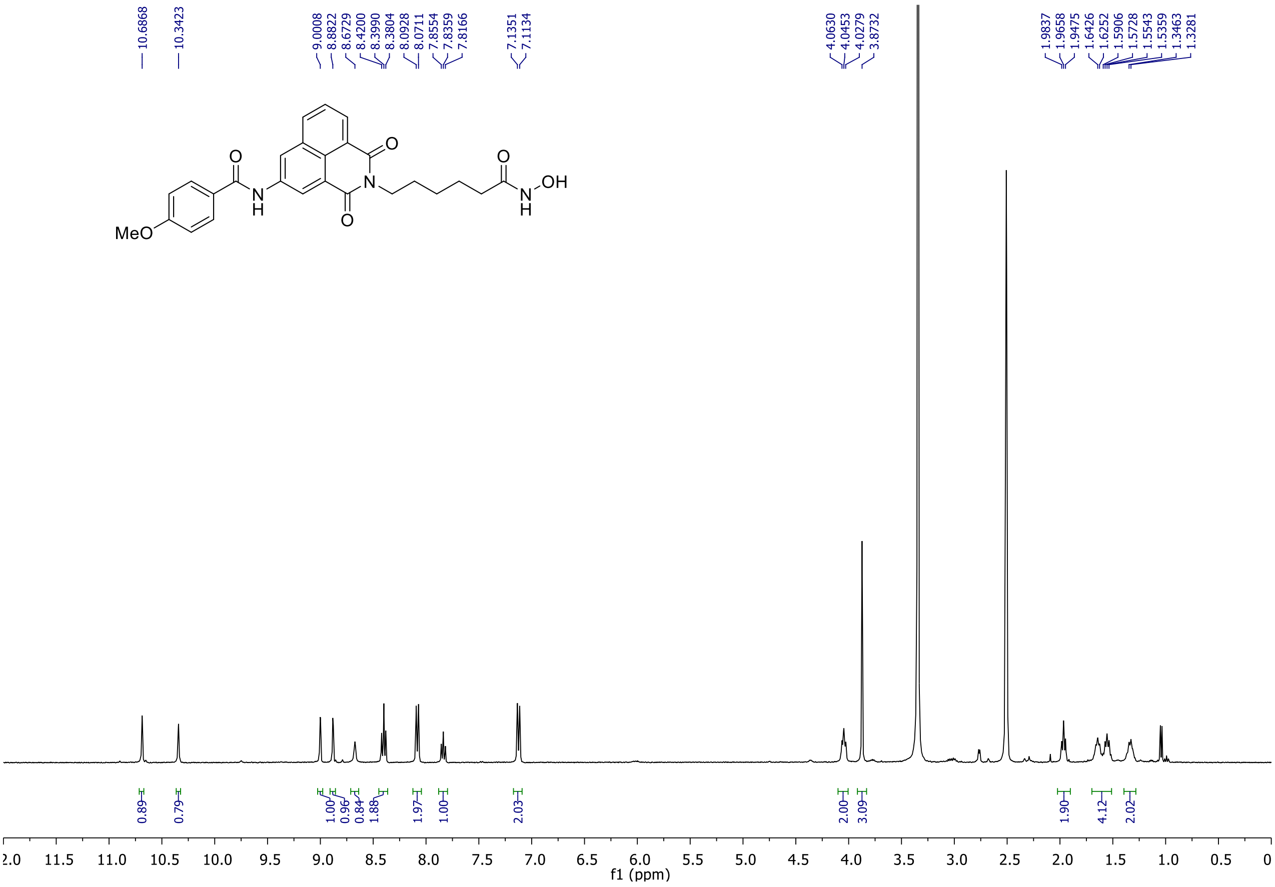

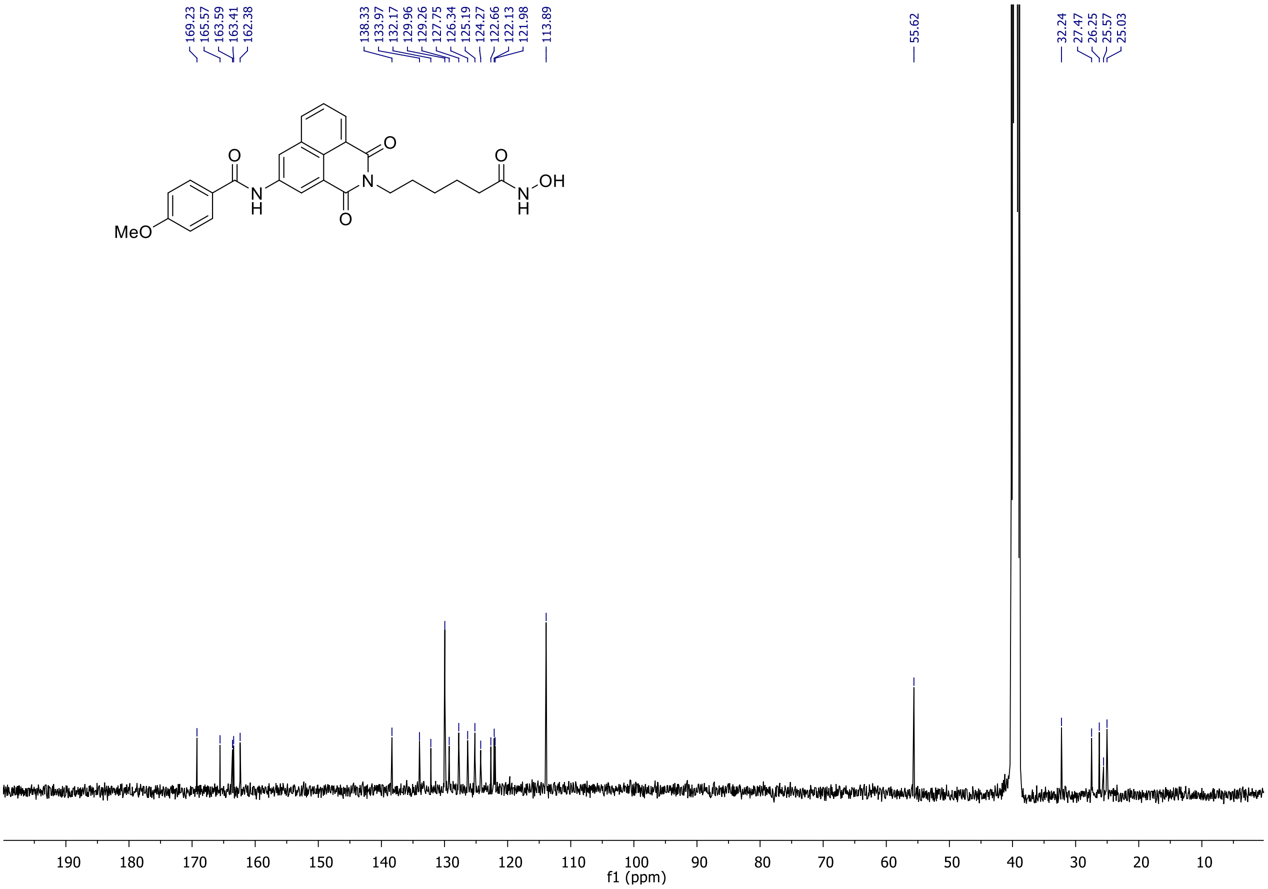

Figure S14 – ^{1}H and ^{13}C NMR of **16**

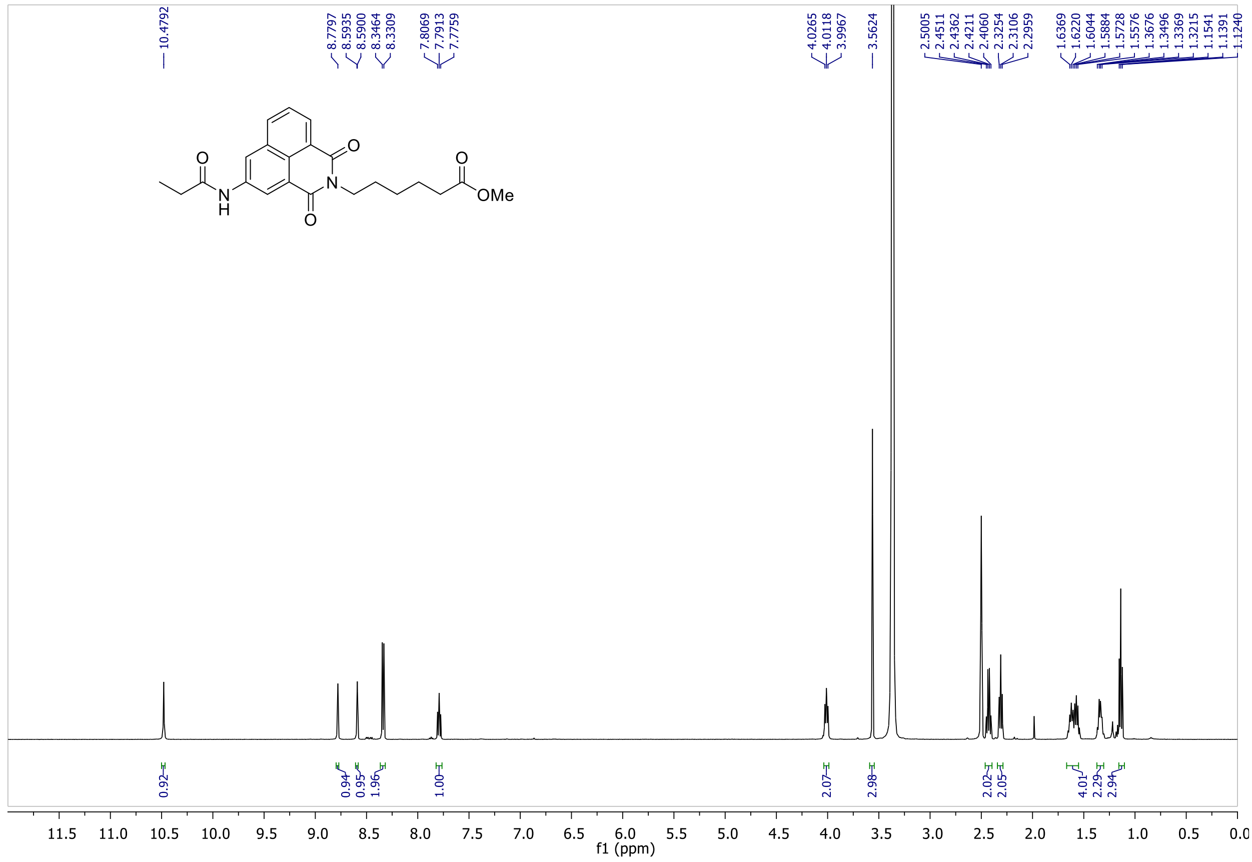

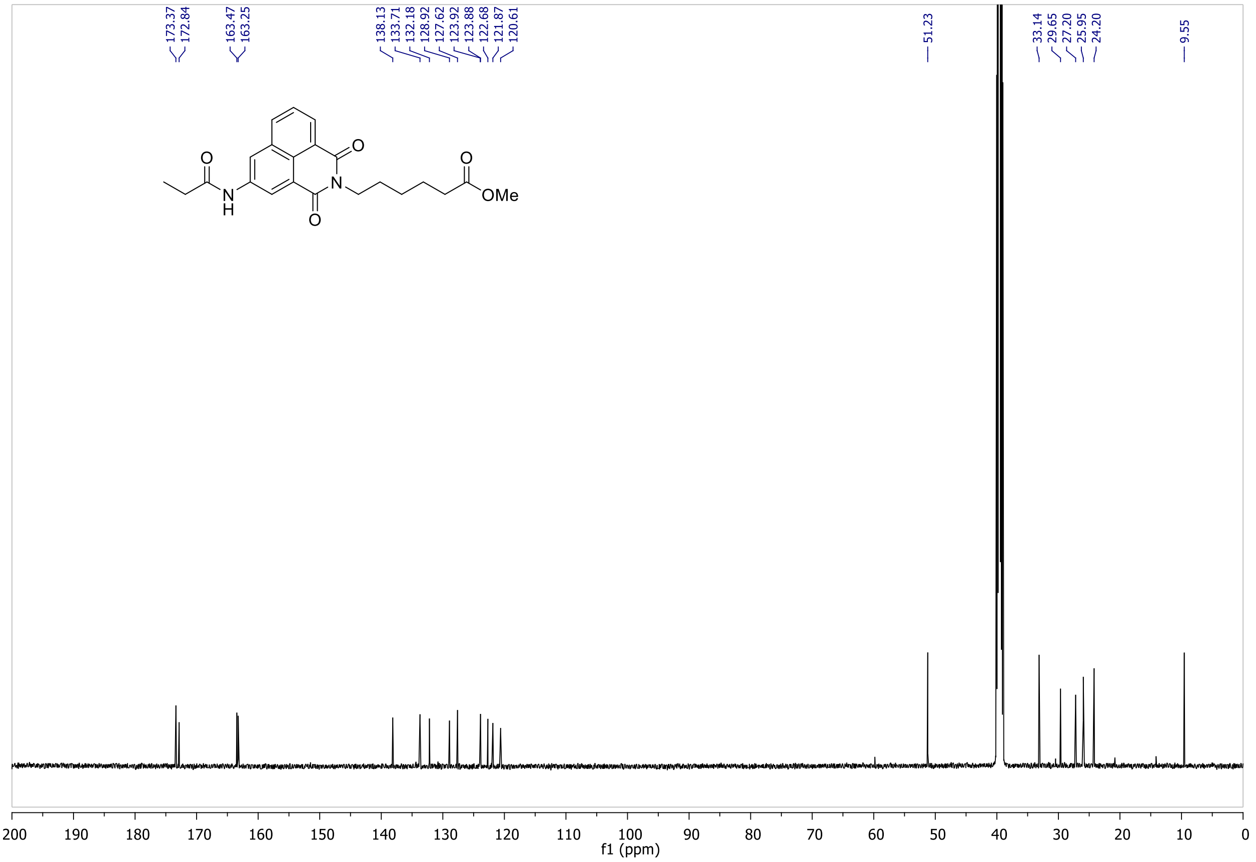

Figure S15 – ^{1}H and ^{13}C NMR of **17**

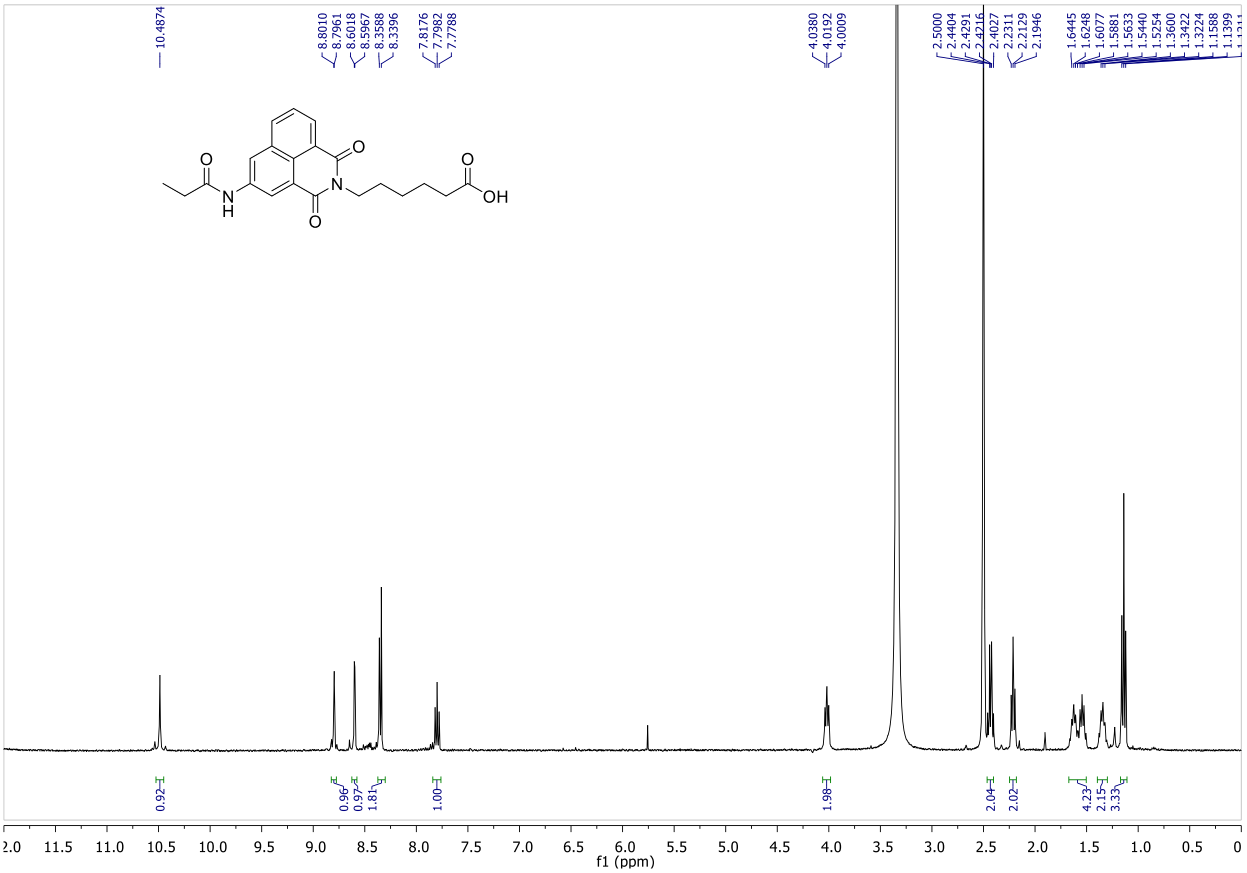

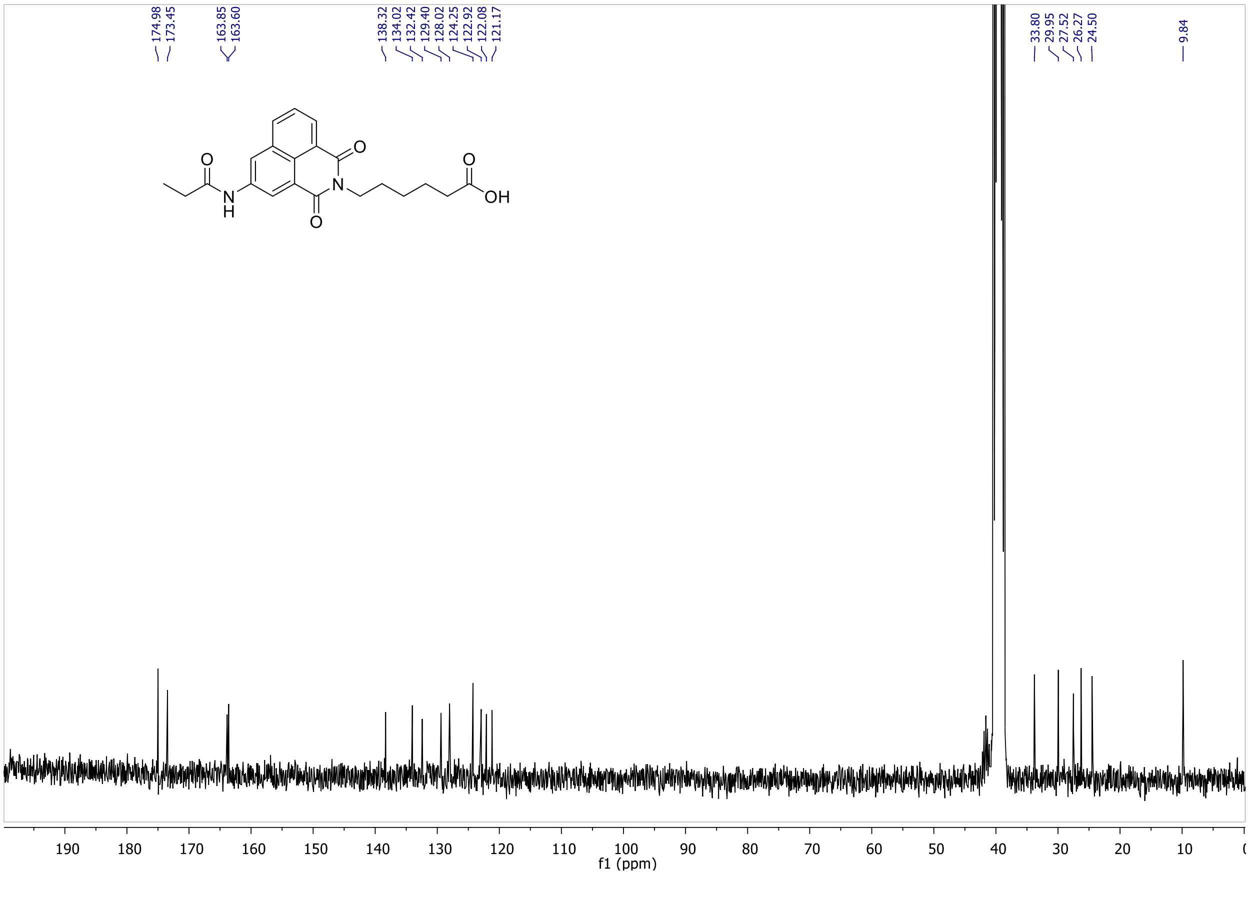

Figure S16 – ^{1}H and ^{13}C NMR of **KNH021**

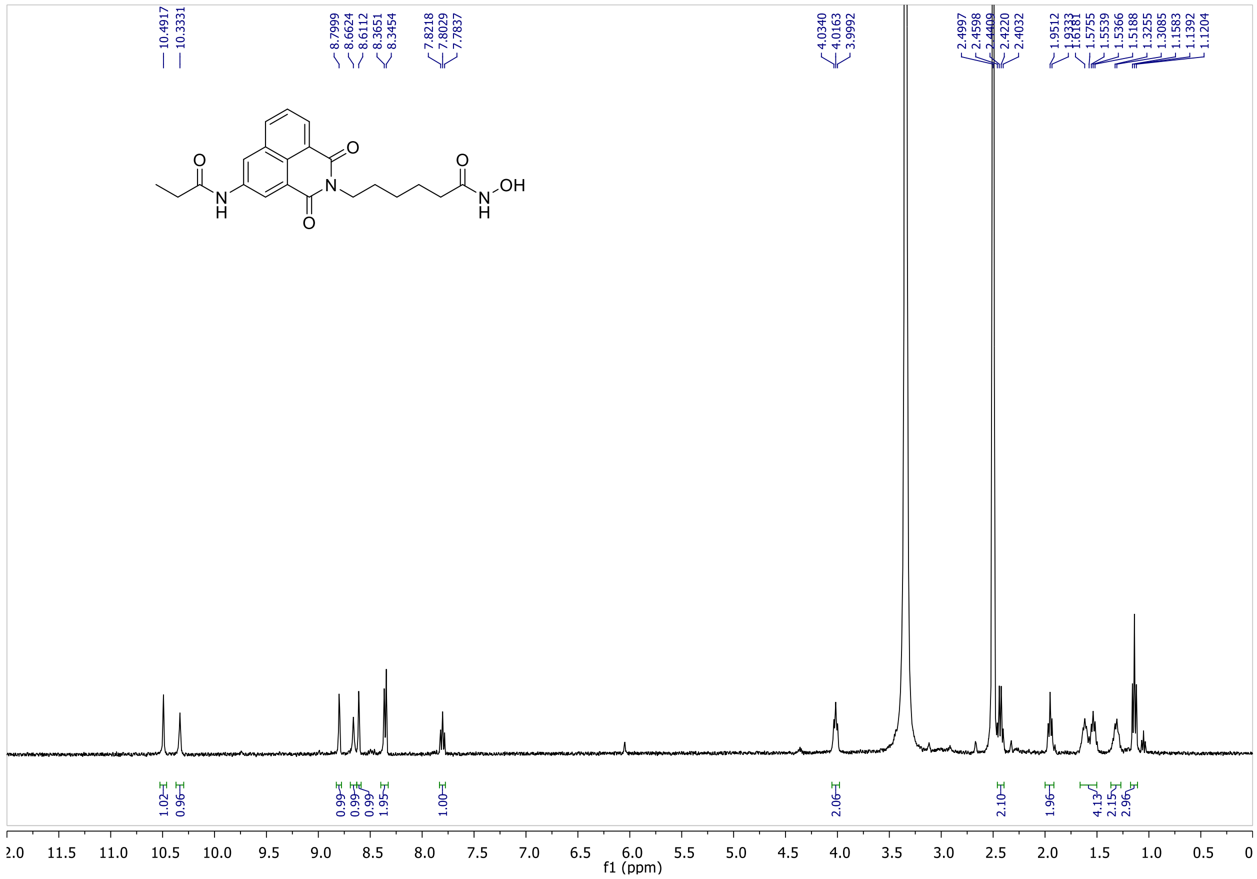

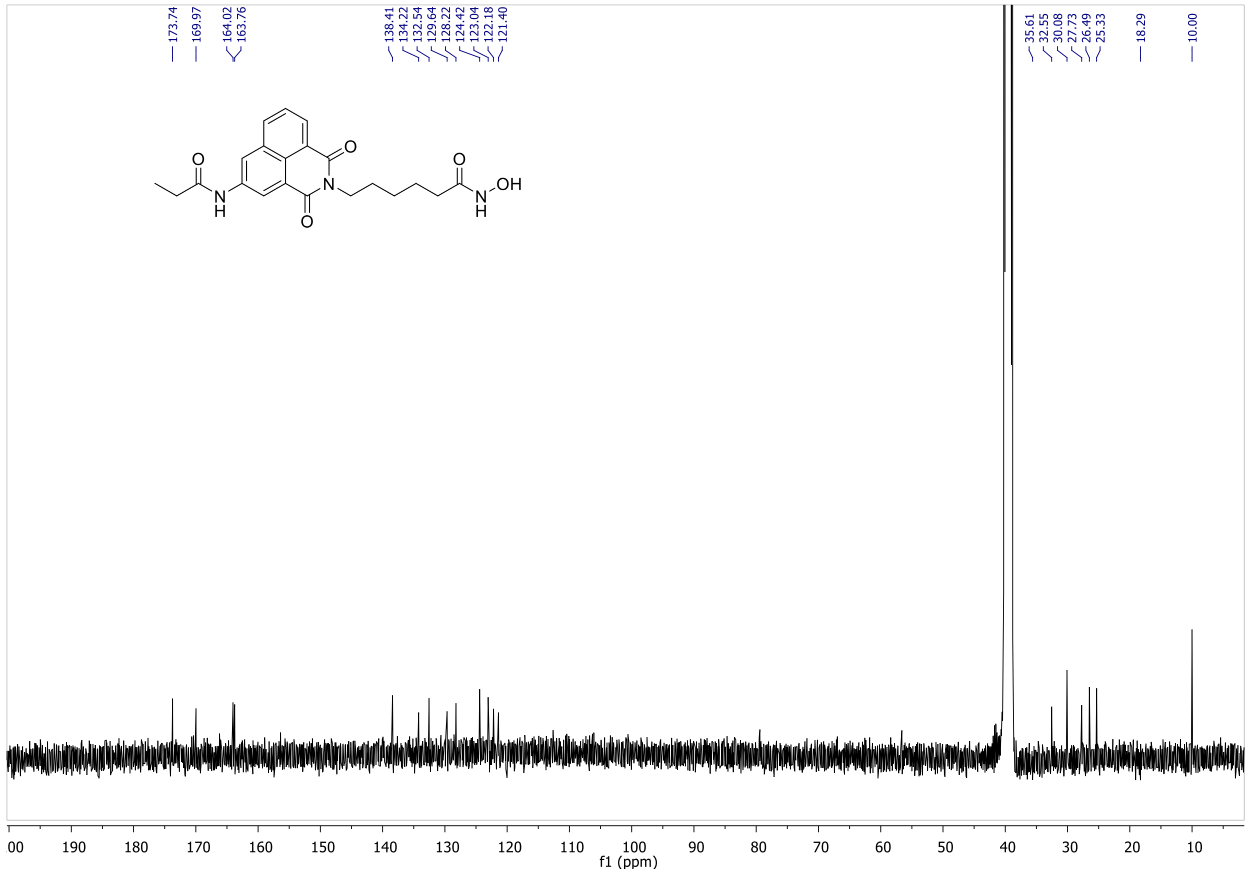

S1.6 UV/Vis and Fluorescence Data

UV/Vis analysis was performed on a Cary Eclipse 300 UV/Vis spectrophotometer and fluorescence measurements performed on a Cary Eclipse Fluorescence spectrophotometer. All analysis was performed in 10 mm quartz cuvettes using AR grade DMSO. Relative quantum yields were obtained by comparison to quinine sulfate ($\phi_f = 0.58$ in 0.1 M H_2SO_4) with absorbance of samples adjusted so that the absorbance at 340 nm was similar to the reference. Emission spectra were obtained by excitation at 340 nm. Quantum yield was obtained by use of the following equation and are reported as the average of two samples with error <5%;

$$\phi_s = \phi_{ref} * \frac{\eta_s^2}{\eta_{ref}^2} * \frac{I_s}{I_{ref}}$$

Where ϕ_{ref} is the literature quantum yield of the reference, η is the refractive index of the solvent (H_2O = 1.333, DMSO = 1.479) and I_s is the integrated fluorescence intensity of the sample with I_{ref} being the same of the reference.

Table S1 – Photophysical properties of selected compounds in DMSO

			Stokes shift		
**Compound**	λ_{abs}	λ_{em}	***(nm)***	***cm^{-1}***	Φ_f [†]
**6**	344	435	91	6081	0.02
	385		50	2986	
**7**	341	439	98	6546	0.04
	383		56	3331	
**8**	343	441	98	6479	0.02
	383		58	3434	
**9**	345	442	97	6361	0.06
	367		75	4623	
**KNH019**	343	437	94	6271	0.05
	374		63	3855	
**KNH020**	343	437	94	6271	0.03
	386		51	3023	
**KNH021**	341	435	94	6337	0.04
	383		52	3121	

[†]Average of two samples, ±1%

Figure S17 – Normalised absorption and emission spectra of **6** in DMSO

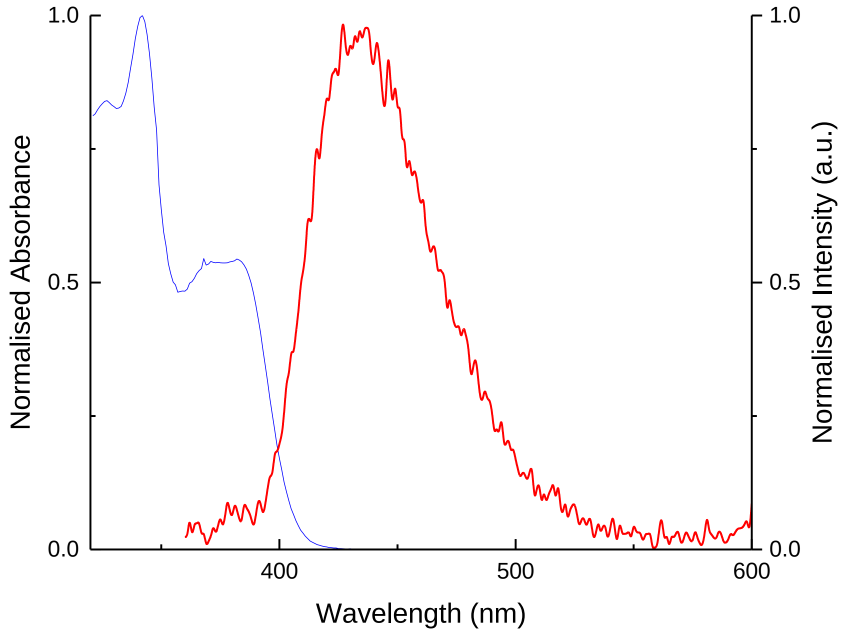

Figure S18 – Normalised absorption and emission spectra of **7** in DMSO

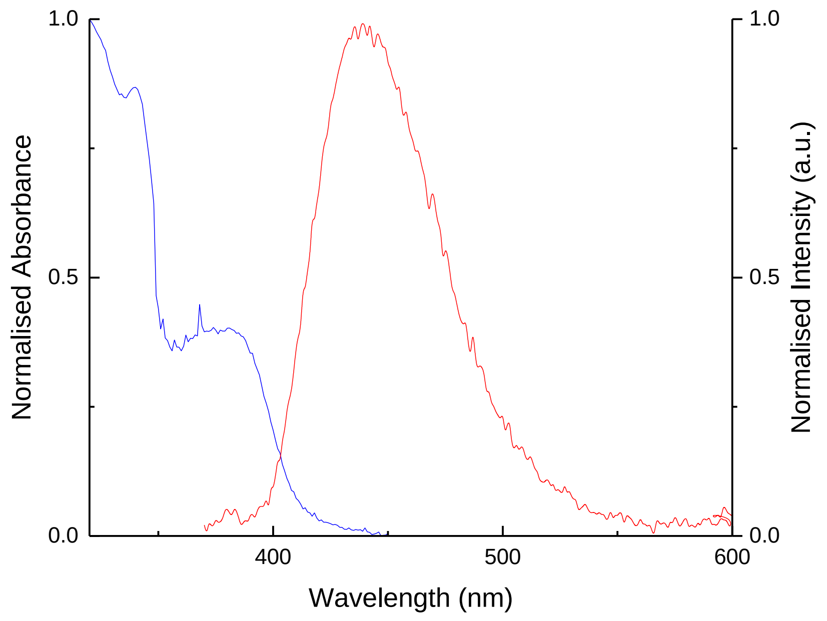

Figure S19 – Normalised absorption and emission spectra of **8** in DMSO

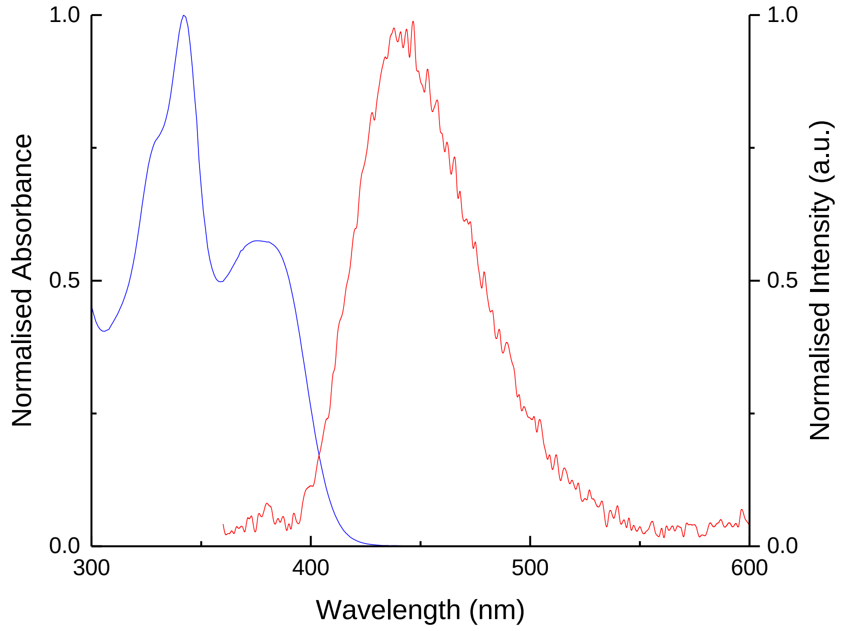

Figure S20 – Normalised absorption and emission spectra of **9** in DMSO

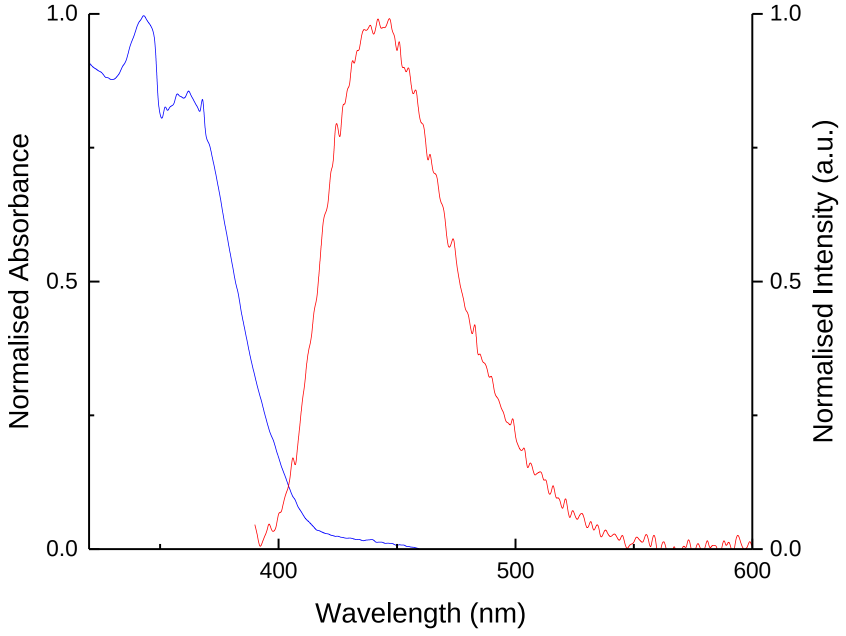

Figure S21 – Normalised absorption and emission spectra of **KNH019** in DMSO

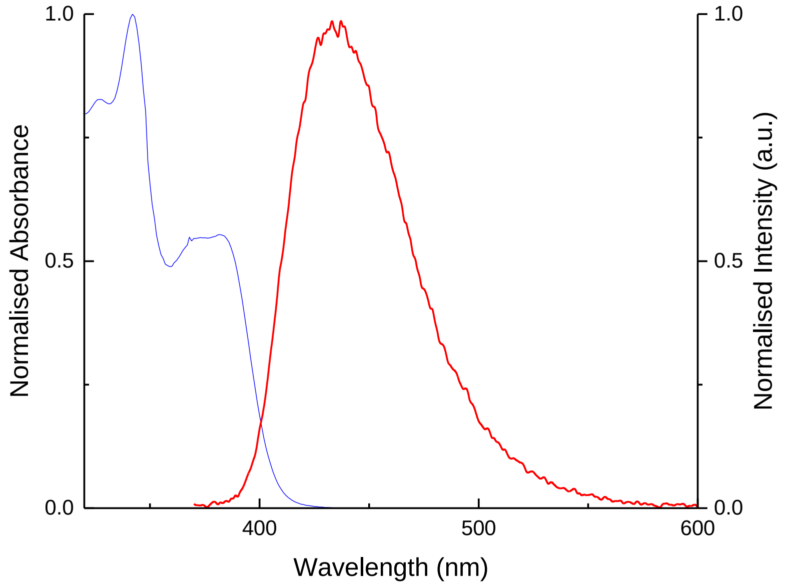

Figure S22 – Normalised absorption and emission spectra of **KNH020** in DMSO

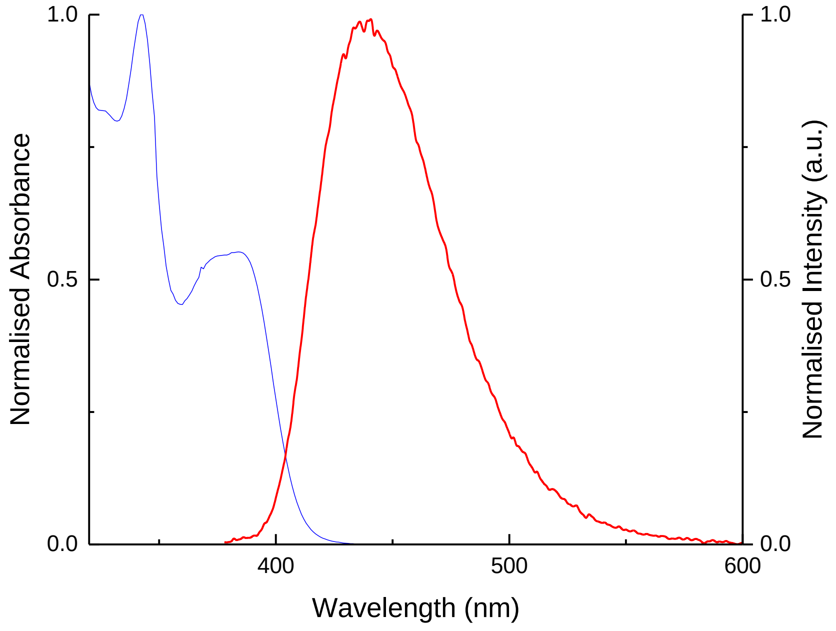

Figure S23 – Normalised absorption and emission spectra of **KNH021** in DMSO

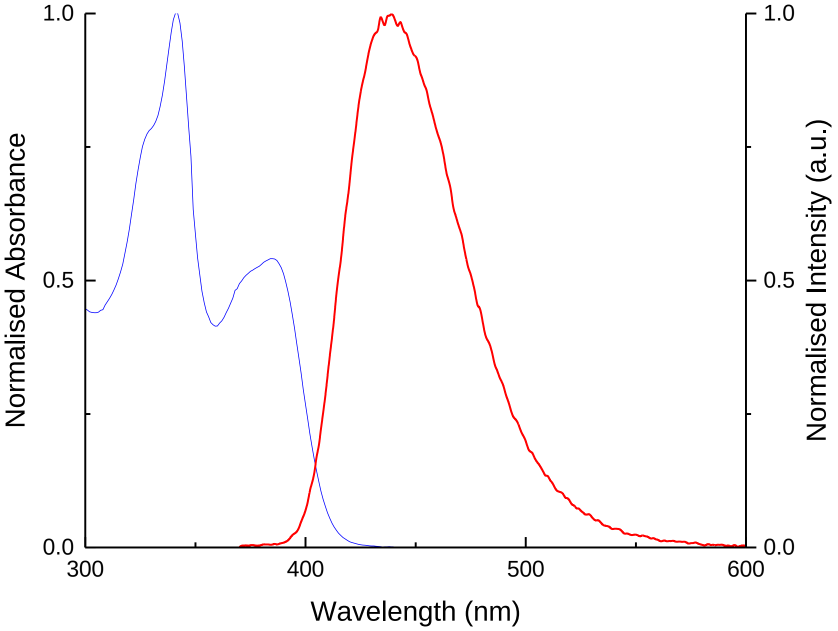

S2 Biology

S2.1 HDAC Assay

All IC_{50} measurements we performed by Reaction Biology corporation. Compounds were tested in either (i) duplicate 10-dose IC50 mode with 3-fold serial dilution starting at 100 uM against HDACs 1, 3, 8 and 11 or (ii) duplicate 10-dose IC50 mode with 3-fold serial dilution starting at 1 uM against HDAC6. HDAC reference compound Trichostatin A (TSA) was tested in a 10-dose IC50 with 3-fold serial dilution starting at 1 uM. Substrate for HDAC1,3,6: 10 uM Fluorogenic peptide from p53 residues 379-382 (RHKK(Ac)AMC). Substrate for HDAC11: 50 uM Fluorogenic HDAC Class2a Substrate (Trifluoroacetyl Lysine). Substrate for HDAC8: 100 uM Fluorogenic peptide from p53 residues 379-382 (RHK(Ac)K(Ac)AMC). IC50 values were calculated using the GraphPad Prism 4 program based on a sigmoidal dose-response equation.

S2.2 Cell Culture, Treatment and Immunostaining:

A549 cells (ATCC® CCL-185™, In Vitro Technologies Pty. Ltd., Victoria, Australia) were cultured in low glucose Dulbecco's Modified Eagle Media (DMEM, 1 g/L glucose, 0.584 g/L L-glutamine, 3.7 g/L sodium bicarbonate, 10 % fetal bovine serum) under standard conditions (> 90 % humidity, 37 °C temperature, 5 % CO_2). For experiments, 5,000 cells/ well were seeded in 100 µl serum free DMEM in 384 well plates (µCLEAR®, Greiner) and left to adhere for 24 hours before the treatment. Cells were treated with 100 µl of test compound-containing media for up to 24 hours before cells were fixed with 4 % paraformaldehyde (PFA) solution in PBS (10 minutes at room temperature (RT)) and permeabilized with 50 µl of 0.5 % Triton X-100 in PBS. Subsequently, cells were blocked in PBST (5% fetal bovine serum, 5% bovine serum albumin, 5% goat serum, 0.1% Tween 20 in PBS) before stained with anti-acetylated tubulin (T7451, Sigma Aldrich; 1:1,000) and anti-acetylated histone antibody (ab47915, Abcam; 1:1,000) overnight at 4 °C. Subsequently, cells were exposed to secondary antibodies (goat anti-mouse Alexa fluor 594 (Invitrogen; 1:10,000) and goat anti-rabbit Alexa fluor 647 (Invitrogen; 1:10,000) in PBST) and DAPI solution (Invitrogen; 1:10,000 in PBST) before images were acquired.

S2.3 Image Analysis:

Fluorescence images were acquired (10x magnification, 2-D format) using the InCell 2200 high content imaging system (V7.3, GE Healthcare life sciences) with CY3 (excitation: 542/27 nm, emission: 597/45 nm), CY5 (excitation: 632/22 nm, emission: 684/25 nm) and DAPI (excitation: 390/18 nm, emission: 435/48 nm) filters. Two images/well at predefined positions were automatically acquired with 4 replicate wells for each treatment condition/ experiment. At least 3 independent experiments were conducted for each condition. Acquired images were automatically processed using InCarta software (v1.6, GE Healthcare life sciences). All images were manually checked for artefacts. Images with less than 30 cells were excluded from the analysis. Excel and GraphPad Prism was used for statistical analysis and graphical representation. Data was expressed as % Relative Fluorescence Units (RFU) compared to the untreated cells, and graphically presented as mean ± standard error of mean (SEM).

S2.4 Cell-based Assessment of HDAC Activity and Selectivity in HepG2 Cells.

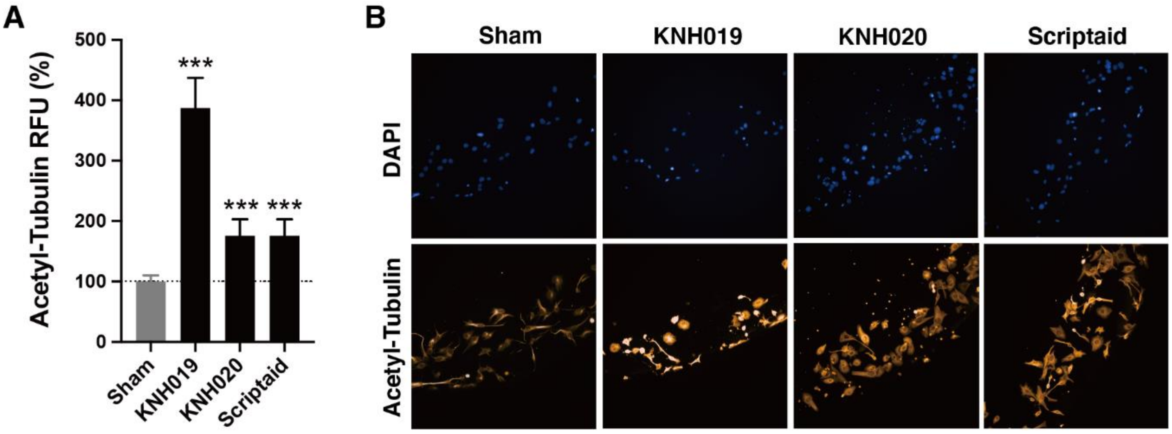

Figure S24: A) HepG2 cells were treated with test compounds (10 µM) for 24 hours (A, B) before tubulin acetylation was automatically quantified using high content imaging. Data represent the average of 2 independent experiments with 4 replicate wells/experiment. Statistical significance of effects compared to the untreated control were analysed using one-way ANOVA analysis followed by Dunnett’s multiple comparison post-test using Graph Pad Prism. Significance was set as ***p < 0.001. Error bars represent Standard Deviation (SD). **B)** Representative fluorescence images show tubulin acetylation (yellow) and nuclear counterstain (DAPI, blue) after 24h of exposure to test compounds.
